# Cardiotrophin 1 stimulates beneficial myogenic and vascular remodeling of the heart

**DOI:** 10.1038/cr.2017.87

**Published:** 2017-08-08

**Authors:** Mohammad Abdul-Ghani, Colin Suen, Baohua Jiang, Yupu Deng, Jonathan J Weldrick, Charis Putinski, Steve Brunette, Pasan Fernando, Tom T Lee, Peter Flynn, Frans H H Leenen, Patrick G Burgon, Duncan J Stewart, Lynn A Megeney

**Affiliations:** 1 Sprott Centre for Stem Cell Research, Regenerative Medicine Program, Ottawa Hospital Research Institute, Ottawa Hospital, Ottawa, Ontario K1H 8L6, Canada; 2 Department of Cellular and Molecular Medicine, Faculty of Medicine, University of Ottawa, Ottawa, Ontario K1H 8M5, Canada; 3 University of Ottawa Heart Institute, Ottawa, Ontario K1Y 4W7, Canada; 4 Department of Biology, Carleton University, Ottawa, Ontario K1S 5B6, Canada; 5 Fate Therapeutics Inc., 3535 General Atomics Court Suite 200, San Diego, CA 92121, USA; 6 Department of Medicine (Cardiology), Faculty of Medicine, University of Ottawa, Ottawa, Ontario K1H 8M5, Canada

**Keywords:** cardiotrophin 1, cardiac, hypertrophy, right heart failure, physiologic, reversible, caspase

## Abstract

The post-natal heart adapts to stress and overload through hypertrophic growth, a process that may be pathologic or beneficial (physiologic hypertrophy). Physiologic hypertrophy improves cardiac performance in both healthy and diseased individuals, yet the mechanisms that propagate this favorable adaptation remain poorly defined. We identify the cytokine cardiotrophin 1 (CT1) as a factor capable of recapitulating the key features of physiologic growth of the heart including transient and reversible hypertrophy of the myocardium, and stimulation of cardiomyocyte-derived angiogenic signals leading to increased vascularity. The capacity of CT1 to induce physiologic hypertrophy originates from a CK2-mediated restraining of caspase activation, preventing the transition to unrestrained pathologic growth. Exogenous CT1 protein delivery attenuated pathology and restored contractile function in a severe model of right heart failure, suggesting a novel treatment option for this intractable cardiac disease.

## Introduction

Heart muscle growth, commonly referred to as cardiac hypertrophy, is a compensatory response that matches organ size to the systemic demands of the body. Hypertrophy can be a detrimental or beneficial adaptation, depending on the type of growth that occurs. In pathologic hypertrophy, heart muscle mass increases (wall thickness) without a corresponding improvement in function. Pathologic hypertrophy is generally irreversible and readily transitions to heart failure (HF), making this maladaptive process a leading cause of morbidity and mortality^1^. Given the prominence in disease etiology, the biochemical and molecular characteristics of pathologic hypertrophy have been intensely studied and documented^2,3^.

Physiologic cardiac hypertrophy is a form of beneficial remodeling, characterized by a modest increase in heart mass with improved contractile function that is reversible. Both pregnancy and endurance exercise provide well-documented means to engage this form of organ growth, a response that can also directly antagonize pathologic hypertrophy and the progression to HF^4,5^. Akt- and MAPK-mediated signaling cascades appear to be consistent molecular signatures of physiologic hypertrophy, yet there is a paucity of definitive information regarding systemic factors that may initiate or propagate this healthy remodeling event^6,7,8,9,10^. Insulin-like growth factor has been examined as a probable physiologic hypertrophy agonist^11,12,13^, yet the pleiotrophic effects of this hormone may preclude its use as a bona fide cardiac restoration agent.

Cardiotrophin 1 (CT1) was originally identified as a promising hypertrophic agonist *in vitro*^14,15^, however its expression has been more recently linked to myocardial pathology, systemic elevated blood pressure, and cardiac failure in both animals and humans^16,17,18,19^. Despite these observational data implicating CT1 in certain cardiovascular diseases, this cytokine is known to bind and engage gp130 receptor complexes, a known pro-survival signal for cardiomyocytes^20,21,22^. Therefore, we reasoned that elevated expression of CT1 in human cardiac pathologies may simply reflect a compensatory response, which attempts to curtail disease progression through the biologic remodeling activity of CT1.

Here, we demonstrate that human CT1 protein (hCT1) engages a fully reversible form of myocardial growth, and that hCT1 attenuates the ongoing pathology and loss-of-function in an aggressive and unremitting model of right heart failure (RHF). hCT1 promotes cardiomyocyte growth in part by inducing a limited activation of an otherwise pathologic hypertrophy signal, as mediated by the caspase 3 protease. In addition, hCT1 engages a cardiomyocyte-derived vascular growth signal to ensure that the modest heart muscle growth is temporally matched with a supporting angiogenic response. Moreover, 2 weeks administration of hCT1 *in vivo* produced cardiac remodeling that was similar to that induced by exercise and, in a model of progressive RHF due to severe pulmonary arterial hypertension, improved cardiac function and reversed right ventricle (RV) dilatation. These data suggest that hCT1 fulfills the criteria as a beneficial remodeling agent, with a capacity to curtail or limit an intractable form of HF.

## Results

### hCT1 induces fully reversible cardiomyocyte cell growth through serial sarcomere addition

**Figure 1:**
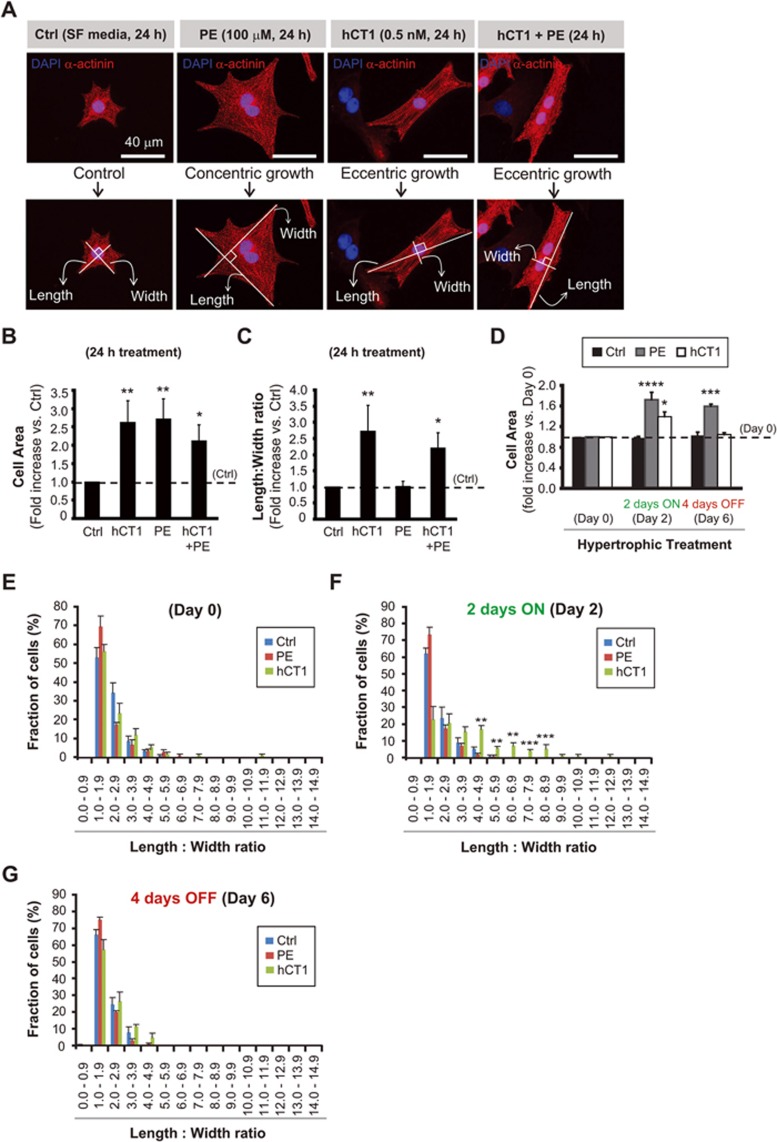
Human cardiotrophin 1 (hCT1) induces morphologic changes in primary cardiomyocytes with reversion upon removal of hCT1 stimulation. **(A)** Primary neonatal cardiomyocytes were stimulated for 24 h with hCT1 (0.5 nM), PE (100 μM), hCT1 and PE, or control serum-free medium (Ctrl, SF) followed by morphometric analysis (cell area and length:width ratio). Cells were stained with α-actinin (red) and nuclei were stained with DAPI (blue). Scale bar, 40 μm. **(B, C)** Morphometric analysis of **(A)** above. Treatment with hCT1, PE, and hCT1+PE induced a significant increase in cell area versus control (*n* = 3; ^*^P < 0.05 and ^**^P < 0.01). However, only hCT1 (or hCT1+PE) stimulation resulted in elongated/eccentric growth versus control (*n* = 3; ^*^P < 0.05 and ^**^P < 0.01). **(D)** Cardiomyocytes were stimulated as in **(A)** above, but for 2 days followed by removal for 4 days. After 2 days, both hCT1 and PE induced hypertrophy versus control at day 2 (*n* = 3; ^*^P < 0.05 and ^****^P < 0.0001). Cells reverted to pre-treatment dimensions upon removal of hCT1, whereas removal of PE caused cells to remain hypertrophied versus Control at day 6 (*n* = 3; ^***^P < 0.001). **(E-G)** Length:width ratio population frequency analysis of **(D)** above. The majority of cardiomyocytes (∼ 60%) display a length:width ratio of 1.0-2.0 (Day 0); however, upon treatment with hCT1 (Day 2), a significant shift in length:width ratio of 3.0-9.0 was observed versus control at day 2 (*n* = 3; ^**^P < 0.01 and ^***^P < 0.001) with reversion occurring at day 6 upon hCT1 removal.

We hypothesized that CT1 may induce physiologic cardiomyocyte growth and beneficial myocardial adaptation. To test this hypothesis we examined whether human recombinant CT1 (hCT1) protein altered the structure of rodent neonatal ventricular cardiomyocytes. We reasoned that if CT1 was a conserved remodeling factor, then the human protein should engage a positive impact on rodent cardiomyocytes. Cardiomyocytes treated with hCT1 displayed a dramatic reorganization in cell architecture within 24 h, leading to a significant increase in the length to width ratio compared to untreated cardiomyocytes (Figure 1A-1C), as was reported previously for CT1^15^, but in contrast to the initial report of the discovery of CT1, which described a concentric growth pattern in treated cells^14^. The increase in the length to width ratio with hCT1 exposure, which was associated with adding sarcomeres in series, was in direct contrast to the morphologic alterations that occurred when cardiomyocytes were treated with pathologic hypertrophy agonists such as the α-adrenergic stimulant phenylephrine (PE) (Figure 1A). PE-treated cells engaged concentric growth, typically characterized by sarcomeres added in parallel, leading to increased cell size, but with a reduced length to width ratio (Figure 1A-1C).

A defining characteristic of physiologic hypertrophy is the fact that the adaptation is fully reversible on cessation of the stress, with individual cardiomyocytes and the intact heart itself returning to pre-stress dimensions^5^. When cardiomyocytes were treated with hCT1 for a 48 h period, followed by removal of the cytokine, the cells rapidly resumed pre-treatment dimensions, similar to untreated cardiomyocytes (Figure 1D-1G). This reversible growth phenotype contrasted with PE-treated cells, where discontinuation of PE did not lead to a decrease in cell size over the same time period, a consistent feature of pathologic hypertrophy induction *in vivo* (Figure 1D-1G).

Aerobic exercise regimes can limit or lessen pathologic cardiac hypertrophy and negative remodeling^5^, suggesting that beneficial growth factors (such as CT1) may similarly impede pathologic inductive cues. Notably, when cardiomyocytes were treated with both PE and hCT1, the cells displayed the growth characteristics of hCT1 alone (Figure 1A-1C). This observation suggests that hCT1 may recapitulate a key aspect of the exercise response, and limit or repress ongoing pathologic growth signals in heart muscle cells.

### hCT1 stimulates a reversible and non-pathologic growth of the intact heart

To date, all extant *in vivo* observational studies of CT1 and heart function have shown negative correlation data, linking elevations in circulating/plasma CT1 to various forms of cardiac dysfunction^18,19,23^. A single study examining exogenous delivery of high concentrations of bolus-injected CT1 protein noted increased gross weight of the heart and morphology consistent with pathologic hypertrophy^17^. However, carefully monitored dose-response studies in both mouse and pig have shown that administration of CT1 protein protects the liver against ischemia reperfusion injury^24,25^, while other studies have reported that chronic CT1 protein delivery augments fatty acid metabolism and reduces insulin resistance^26^, all of which suggest that CT1 conveys a beneficial systemic response.

**Figure 2:**
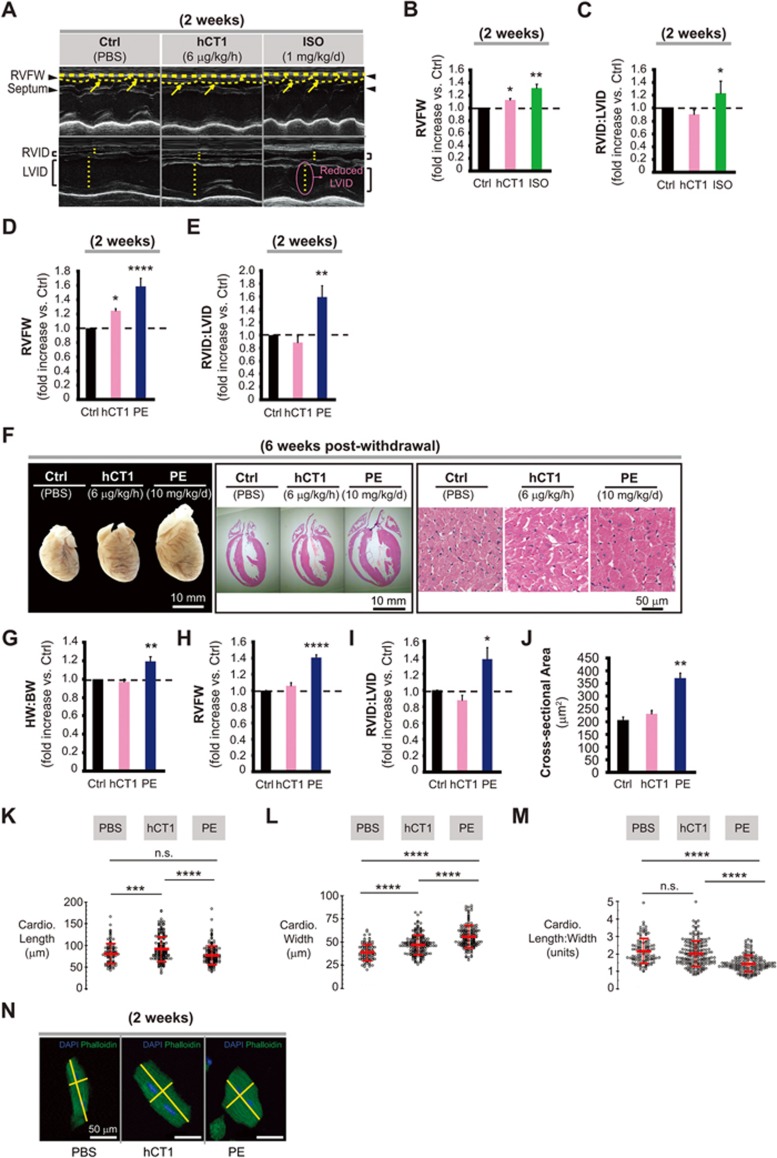
Administration of hCT1 *in vivo* induces beneficial cardiac growth, while withdrawal of hCT1 causes reversion to baseline cardiac dimensions. **(A-J)** Osmotic minipumps were implanted subcutaneously in rats containing PBS control (Ctrl), hCT1 (6 μg/kg/h), and isoproterenol (ISO, 1 mg/kg/d) or phenylephrine (PE, 10 mg/kg/d) to induce hypertrophy over 2 weeks. After 2 and 6 weeks post treatment, M-mode echocardiography analysis was used to assess myocardial structure/function. **(A)** Echocardiography images. Arrows and arrowheads (upper panels) point to the right ventricle free wall (RVFW) and septum. Dotted lines (lower panels) span the right and left ventricle internal diameter (RVID and LVID, respectively) of the inner chambers. **(B-E)** Both hCT1 and ISO (or PE) induced a significant increase in cardiac hypertrophy versus control (*n* = 6; ^*^P < 0.05, ^**^P < 0.01 and ^****^P < 0.0001). However, only rats treated with ISO or PE exhibited an increase in the RVID:LVID ratio (*n* = 6; ^*^P < 0.05 and ^**^P < 0.01). **(F-J)** 6 weeks post withdrawal, hCT1-treated rats displayed reversion of heart growth, whereas PE-treated rats maintained a significant increase in HW:BW (heart weight to body weight ratio), RVFW wall thickness, cardiomyocyte cross-sectional area, and RVID:LVID ratio versus control (*n* = 6; ^*^P < 0.05, ^**^P < 0.01 and ^****^P < 0.0001). **(F)**, Whole hearts (left panel; scale bar, 10 mm), Hematoxylin and Eosin-stained sections (middle and right panels; scale bars, 10 mm and 50 μm, respectively). **(K-N)** Similar procedure as in **(A)** above, however, adult murine cardiomyocytes were analyzed. After 2 weeks of treatment, hCT1 significantly increased the length of cardiomyocytes versus PBS control or PE (**K**; *n* = 6, ^***^P < 0.001 and ^****^P < 0.0001) while PE significantly increased the width versus PBS control and hCT1 (**L**; *n* = 6, ^****^P < 0.0001). The length to width ratio of PE-treated cardiomyocytes was significantly decreased versus PBS control and hCT1 (**M**; *n* = 6, ^****^P < 0.0001), whereas no significant difference (ns) was observed between PBS control and hCT1. Representative images **(N)** of murine cardiomyocytes were stained with Alexa Fluor-488 Phalloidin (F-actin, green) and DAPI (nuclei, blue). Scale bar, 50 μm.

Given these considerations, we chose to examine whether sustained 2 weeks delivery of hCT1 was beneficial or harmful to cardiac morphology and function. The decision to test 2 weeks of protein delivery was predicated on the hypothesis that the capacity of a biologic agent (hCT1 protein) to engage cardio-adaptive measures would most likely require sustained exposures, akin to the systemic changes which occur during exercise and pregnancy. hCT1-loaded osmotic minipumps were surgically implanted into rodents and protein was delivered for a continuous 14-day period (at 6 μg/kg/h, a hCT1 concentration within the dose range noted above). Animals subjected to this hCT1 dosing regimen displayed a cardiac remodeling that was reminiscent of exercise adaptation, with a modest yet significant increase in ventricle wall thickness and no alteration in right to left ventricle internal diameter ratio (RVID:LVID) (Figure 2A-2C). Different alterations were observed in isoproterenol (ISO) or PE-treated animals, which displayed a dramatic increase in wall thickness, with concomitant severe change in the RVID:LVID ratio (Figure 2B-2E). Importantly, we noted a complete reversion of cardiac growth/remodeling at 6 weeks following discontinuation of hCT1 after a 2-week exposure. Here, the overall heart mass of hCT1-treated animals was similar to control animals, with ventricle wall thickness and RVID:LVID ratios reverting to baseline levels (Figure 2F-2I). However, animals treated with PE for a similar 2-week period, followed by 6 weeks of recovery, continued to display a cardiac morphology that is consistent with a pathologic state, i.e., elevated wall thickness and RVID:LVID ratios (Figure 2F-2I). Moreover, changes in myocardial wall thickness were paralleled by a corresponding change in cardiomyocyte cross-sectional area (Figure 2F and 2J), consistent with the CT1-inspired *in vitro* adaptations.

We next examined isolated cardiomyocyte morphology from adult rodent (murine) hearts, which is representative of conditions where true physiologic adaptation occurs. We treated adult mice for 2 weeks with phosphate-buffered saline (PBS), hCT1 (6 μg/kg/h) or PE (10 mg/kg/d), observing that hCT1 caused an increased lengthening of isolated mouse cardiomyocytes versus PE or PBS control (Figure 2K-2N). Furthermore, the length to width ratio of PE-treated cardiomyocytes was significantly less than those for both hCT1 and PBS control, where hCT1 and PBS treatments resulted in similar length to width ratios (Figure 2K-2N). These data are similar to what we observed for isolated and cultured neonatal primary cardiomyocytes (Figure 1), the only notable difference is that in the *in vitro* model there was a significant increase in the length to width ratio and not just length following hCT1 treatment, as was the case in the *in vivo* assessments (Figure 2K-2N). The greater length changes *in vitro* with hCT1 administration may be attributed to a lack of physical constraints within this system, which allows the cells to continue to add sarcomeres in series, beyond what physical space constraints would be permissive *in vivo*.

### hCT1 stimulates beneficial cardiomyocyte remodeling through a constrained activation of caspase 3

The morphologic variation in ventricular cardiomyocytes during pathologic versus physiologic hypertrophy suggests that distinct signaling mechanisms control these divergent cell adaptations. This is a generally accepted hypothesis and robust evidence exists to support this proposition, i.e., activation of Akt-mediated signals induces a physiologic hypertrophy and offers protection against pressure overload pathology, while calcineurin-mediated signals are largely regarded as pathologic growth cues^3,8^. The primary limitation with this concept is that the early stages of pathologic growth are often compensatory or beneficial in nature in response to a stress adaptation^1^. As such, a reasonable nuance to the prevailing hypothesis is that the divergent forms of hypertrophy may share a common molecular origin, where the more extreme form of growth (pathologic hypertrophy) derives from an unconstrained molecular signal. For example, the Mef2 transcription factor family has been noted to have elevated activities in both pathologic and maturational cardiac growth programs^27,28,29^.

**Figure 3:**
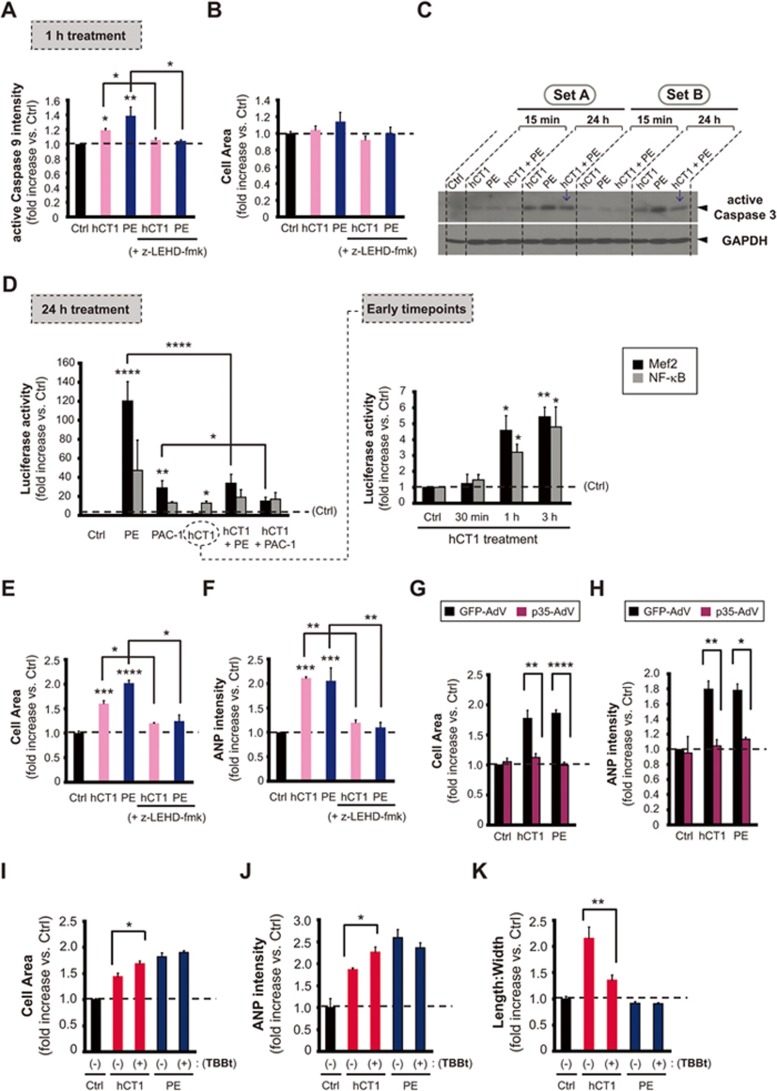
hCT1 engages a restricted activation of the intrinsic caspase-mediated cell death pathway. **(A, B)** Primary cardiomyocytes were treated for 1 h with control serum-free medium (Ctrl), hCT1 (0.5 nM), or PE (100 μM) in the presence or absence of the caspase 9 inhibitor (z-LEHD-fmk, 20 μM). Caspase 9 was significantly activated with hCT1 and PE versus control (*n* = 3; ^*^P < 0.05 and ^**^P < 0.01) and this was decreased in the presence of z-LEHD-fmk (*n* = 3; ^*^P < 0.05). **(C)** Cardiomyocytes were treated with hCT1 (0.5 nM), PE (100 μM), or hCT1+PE for 15 min and 24 h and analyzed by western immunoblotting. hCT1 caused a moderate increase in caspase 3 activity compared to PE at 24 h. Caspase 3 activity also decreased with combined PE and hCT1 stimulation (arrows). GAPDH was the loading control. **(D)** Cardiomyocytes were transfected with reporter plasmids under the control of NF-κB or Mef2 promoters and luciferase activity was measured after treatment with: control serum-free medium (Ctrl), hCT1 (0.5 nM), PE (100 μM), or procaspase 3-activating compound 1 (PAC-1; 25 μM). hCT1 treatment resulted in NF-κB and Mef2 activation at 1 and 3 h versus control (*n* = 4; ^*^ *P* < 0.05 and ^**^P < 0.01); and at 24 h, only NF-κB activity was sustained (*n* = 4; ^*^P < 0.05). However, PE and PAC-1 treatment caused significant activation of Mef2 after 24 h (*n* = 4; ^**^P < 0.01 and ^****^P < 0.0001). Co-stimulation of hCT1/PE and hCT1/PAC-1 significantly reduced Mef2 activity compared to PE and PAC-1 alone (*n* = 4; ^****^P < 0.0001 and ^*^P < 0.05, respectively). **(E, F)** Similar procedure as in **(A)** above; however, with 24 h treatment. hCT1 and PE significantly increased cell area and the pro-hypertrophic marker ANP versus control (*n* = 3; ^***^P < 0.001 and ^****^P < 0.0001) and this was significantly attenuated in the presence of z-LEHD-fmk (*n* = 3;*P < 0.05 and ^**^P < 0.01). **(G, H)** Cardiomyocytes were infected for 24 h with an adenovirus (AdV) encoding the caspase inhibitor, p35-AdV, prior to inducing hypertrophy with hCT1 or PE for 24 h. GFP-AdV was used as a control. p35-AdV significantly inhibited hCT1 and PE induced hypertrophy (*n* = 3; ^**^P < 0.01 and ^****^P < 0.0001, respectively) and inhibited ANP expression (*n* = 3; ^**^P < 0.01 and ^*^P < 0.05, respectively). Both p35-AdV and GFP-AdV were used at a mean of infectivity (MOI) of 20. **(I-K)** Similar procedure as in **(E, F)** above; however, casein kinase 2 (CK2) activity was blocked using TBBt (50 μM). CK2 inhibition significantly increased cell size and ANP expression (*n* = 3; ^*^P < 0.05) while reducing length:width ratio (*n* = 3; ^**^P < 0.01) when compared to hCT1 treatment alone.

To address this supposition we examined whether hCT1-induced cardiomyocyte remodeling was associated with a blunted activation of the mitochondrial-/intrinsic-mediated cell death pathway. This sequential proteolytic cascade was initially defined as a hallmark of apoptosis, yet this pathway is now widely recognized as a signaling cascade that confers a variety of essential non-death cell adaptations^30,31,32^. Prior observations from our laboratory have demonstrated that agonists producing pathologic hypertrophy engage robust activation of the intrinsic cell death pathway and distal pathway activation alone (caspase 3) is essential and sufficient to induce hypertrophy without attendant apoptosis^33^. We noted that hCT1 treatment of cardiomyocytes resulted in a rapid proximal activation of this signal cascade, with increased caspase 9 activity (Figure 3A-3B; Supplementary information, Figure S1A). Caspase 3 activity was effectively engaged by hCT1, yet was notably decreased when compared with that in the presence of the hypertrophy agonist PE, with a similar reduction in caspase 3 activity when cardiomyocytes were treated with both hCT1 and PE (Figure 3C). hCT1-treated cardiomyocytes also rapidly engaged caspase 3-sensitive transcriptional responses, including the activation of Mef2- and NF-κB-dependent reporters (Figure 3D). However, unlike exposure to standard pathologic agonists such as PE and ISO, the activation of Mef2 and NF-κB in response to hCT1 was limited in duration (Figure 3D). hCT1 also selectively engaged a robust STAT3-dependent reporter activity unlike PE, which preferentially activated STAT1 (Supplementary information, Figure S2A and S2B). Interestingly, STAT1 and STAT3 appear to have opposing roles in regulating survival and inflammation: STAT1 acts as a pro-inflammatory factor and induces apoptosis, whereas STAT3 is anti-inflammatory and promotes cell survival^34^. It is the intricate balance between STAT1 and STAT3 activation that may determine the outcome of PE or hCT1 treatment. In this case, hCT1 stimulation is likely cardioprotective due to preferential activation of STAT3 over STAT1 (Supplementary information, Figure S2A and S2B). Finally, blockade of caspase 9 activity (through exposure to peptide inhibitors) or caspase 3 activity (through adenoviral delivery of the biologic effector caspase inhibitor p35) completely abrogated hCT1-induced cell growth and hCT1-induced upregulation of pro-hypertrophic markers such as ANP (Figure 3E-3H; Supplementary information, Figure S1B and S1C). ANP expression increases in both pathologic and physiologic hypertrophy settings, as such production and secretion of this natriuretic peptide provides a robust measure of a global hypertrophy response^35^. Although we provide evidence that caspase 9 and 3 activity are required for hypertrophy induction by both hCT1 and PE, the extent of caspase activity is not as robust during hCT1 stimulation when compared to PE. Importantly, this limited caspase activation in response to hCT1 is still significant enough to engage a beneficial hypertrophic response that is sensitive to caspase inhibition.

These observations suggest that hCT1 engages a restricted activation of the intrinsic cell death pathway to initiate the beneficial remodeling of cardiomyocytes. There are numerous examples in the literature regarding concurrent signals or feedback mechanisms that limit caspase 3 activation and we surmised that such a pathway was operant during hCT1-induced hypertrophy. Here, we examined whether hCT1 was coincident with the activation of CK2, a kinase that phosphorylates both caspase 3 and its cognate substrates, effectively squelching protease activity and limiting proteolytic cleavage^36,37^. Inhibition of CK2 (via exposure to TBBt) during hCT1 stimulation of cardiomyocytes resulted in a significant increase in cell size and ANP expression when compared to cardiomyocytes treated with hCT1 alone (Figure 3I-3J; Supplementary information, Figure S3). Treatment of cardiomyocytes with both PE and the CK2 inhibitor did not lead to any additional increase in cell size or ANP expression compared to PE alone, suggesting that CK2 activation is not a feature of pathologic signaling cascades. The inhibition of CK2 activity during hCT1 stimulation also resulted in a morphologic alteration, where the affected cardiomyocytes displayed a growth phenotype more characteristic of exposure to pathologic agonists such as PE (i.e., a reduction in the length to width ratio, Figure 3K; Supplementary information, Figure S3). These observations imply that hCT1 mediated a constrained activation of caspase 3 activity via CK2-targeted phosphorylation and that this biochemical step is a key feature of beneficial cardiomyocyte remodeling, instrumental in preventing a transition to fulminant pathologic hypertrophy.

### hCT1 stimulates a cardiomyocyte-derived paracrine signal to enhance angiogenesis and limit fibrosis

Physiologic cardiac hypertrophy is associated with an increase in capillary density, whereas pathologic hypertrophy is characterized by a relative decline or no change at all^9,38^. Indeed, this compensatory vascular growth/angiogenesis is permissive for the increased workload that accompanies physiologic hypertrophy. The intra-dependence of these tissue adaptations suggests that the myocardium and its supporting vasculature may engage in a paracrine or autocrine signal to match myocardial growth with an improved blood supply. In support of this premise, vascular endothelial growth factor (VEGF) has been demonstrated to be produced and secreted by cardiomyocytes and gene targeted deletion of this growth factor leads to prominent cardiac pathology^39,40^. Alternatively, a beneficial remodeling agent may act concurrently but independently on myocardial and vascular cells to spur organ growth.

**Figure 4:**
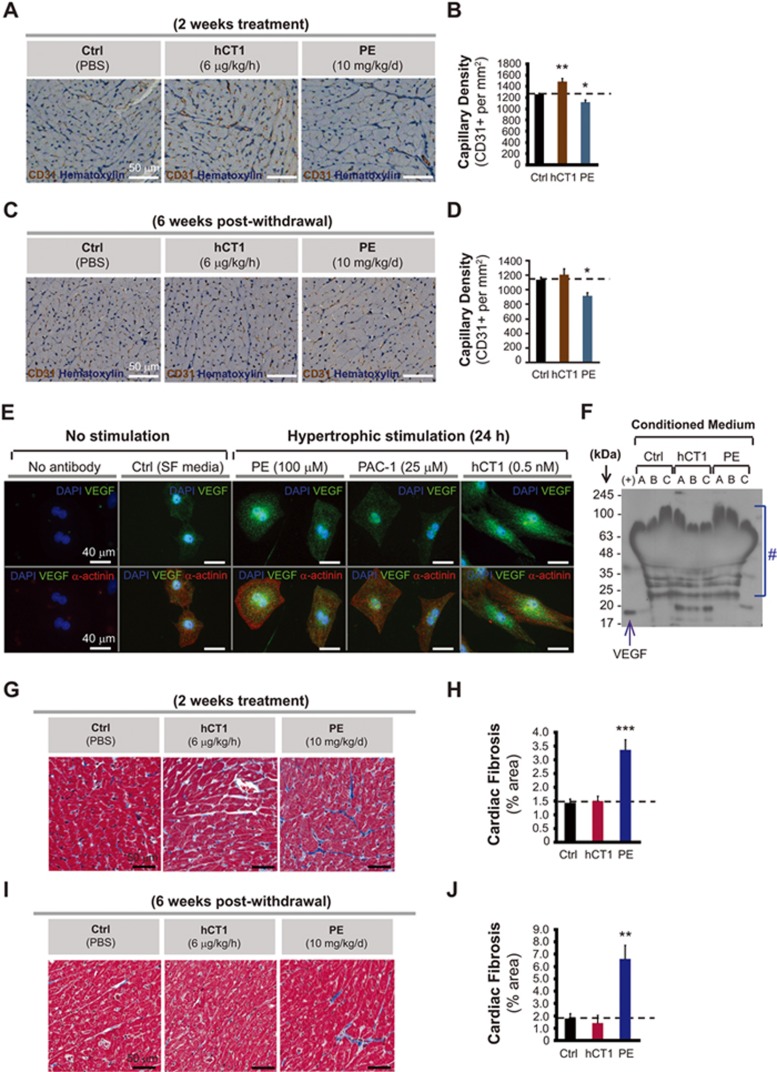
hCT1 enhances angiogenesis and limits fibrosis during cardiac hypertrophy. **(A-D)** Rats were treated with hCT1 or PE for 2 weeks followed by withdrawal for 6 weeks. Capillary density was assessed by staining for CD31 (brown) and counterstaining with Hematoxylin (blue). hCT1-treated rats displayed a significant increase in CD31-positive capillaries (*n* = 4;**P < 0.01) at 2 weeks with reversion at 6 weeks post withdrawal; whereas PE-treated rats displayed a significant decrease at 2 weeks post treatment and 6 weeks post withdrawal (*n* = 4; ^*^P < 0.05). Scale bar, 50 μm. **(E)** Primary cardiomyocytes were incubated with control serum-free medium (Ctrl, SF medium), hCT1 (0.5 nM), PE (100 μM), or PAC-1 (25 μM) for 24 h. hCT1 treatment resulted in a greater increase in vascular endothelial growth factor (VEGF) expression compared to PE or PAC-1. Immunofluorescence was used to detect α-actinin (red), VEGF (green), and nuclei were stained with DAPI (blue). Scale bar, 40 μm. **(F)** Similar procedure as in **(E)** above, however, conditioned medium was harvested at 24 h and expression of secreted VEGF protein was detected upon immunoblotting with anti-VEGF antibody. A significant increase in VEGF was observed in hCT1-treated samples (sets A-C) when compared to PE or Ctrl. (+), recombinant VEGF protein (40 ng) used as positive control. (#), cross-reactivity bands of antibody to components in conditioned medium. **(G-J)** Rats were treated as in **(A)** above and fibrosis/collagen was assessed using Masson's Trichrome staining (blue deposits). Rat hearts exposed to PE exhibited a significant increase in collagen/fibrosis at 2 weeks post treatment as well as 6 weeks post withdrawal (*n* = 4; ^***^P < 0.001 and ^**^P < 0.01, respectively). Scale bar, 50 μm.

To begin to address this supposition we initially examined capillary density in the myocardium of hCT1-treated versus control and PE-treated animals (a similar 14-day treatment regime as noted in Figure 2). hCT1-treated animals displayed a significant increase in the number of CD31-positive capillaries compared to control or PE-treated animals, indicative of beneficial remodeling of the myocardium (Figure 4A and 4B). Discontinuation of hCT1 treatment led to a reversion of capillary density to control values (Figure 4C and 4D), suggesting that the angiogenic properties of hCT1 are fully reversible, akin to hCT1-mediated growth alterations in cardiomyocytes and the myocardium proper (Figures 1 and 2). Next, we sought to identify whether the hCT1 pro-angiogenic activity was associated with a cardiomyocyte-derived paracrine signal. Comparative gene expression analysis revealed that hCT1-treated cardiomyocytes exhibited robust induction of angiogenic factors including angiopoietin-like 4 (Angpt14), Fms-related tyrosine kinase 4 (Flt4), von Willebrand factor 5A (Vwa5a), with modest but significant increases in VEGF expression (Supplementary information, Table S1). Stimulation of cardiomyocytes with hCT1 led to a rapid increase of VEGF protein within the cell, yet treatment with pathologic hypertrophy agonists such as PE or the caspase 3 small molecule activator, PAC-1 resulted in a minimal increase (Figure 4E). In addition, blockade of caspase 3 activity (through adenoviral delivery of the biologic effector caspase inhibitor p35) significantly inhibited the hCT1-induced increase in VEGF, whereas the minimal increase in intracellular VEGF with PE was also inhibited with p35 adenovirus (AdV) (Supplementary information, Figure S4A and S4B). This suggests that VEGF expression induced by hCT1 or PE is mediated, in part, by caspase signaling. In addition, western analysis of media derived from hCT1-treated cardiomyocytes demonstrated that the increased production of VEGF protein was associated with an increased release of the growth factor (Figure 4F). It is interesting to note that the minimal cellular VEGF expression induced by PE was not detected in the media after 24 h (Figure 4F). Presumably, pathologic stimulation with PE impedes any robust secretion of VEGF from the cell thus hindering angiogenesis such that it cannot keep pace with hypertrophy. Indeed, others have detected minor amounts of secreted VEGF in the media after prolonged (48 h) stimulation with PE^41^. These results suggest that hCT1 promotes cardiomyocyte secretion of angiogenic factor(s) via a signaling mechanism that is concurrent to and dependent on caspase 3.

Given that angiogenesis can promote the transport of oxygen and nutrients to cardiomyocytes, we next explored the metabolic effect of inducing hypertrophy with hCT1 or PE. Under normal conditions, the majority of energy (ATP) generated in the heart is derived from mitochondrial oxidative phosphorylation and, during hypertrophy, there is an increased metabolic demand to compensate for the increased workload^42^. Thus, oxygen consumption of cardiomyocytes is an important indicator of normal cellular function where unhealthy cells with dysfunctional mitochondria display a lower oxygen consumption compared to healthy cells and exercise adapted cardiomyocytes display rates of oxygen consumption above baseline values. Accordingly, we measured the rate of oxygen consumption and observed that treatment with hCT1 for 24 h resulted in a significantly higher rate of oxygen consumption compared to pathologic stimulation with PE (Supplementary information, Figure S5). Moreover, the microarray data indicated a slight, yet significant, compensatory decrease at the gene expression level of a wide array of oxidative mitochondrial enzymes (Supplementary information, Table S1). This observation suggests that hCT1 may increase the efficiency of oxidative mitochondrial energy metabolism during physiological hypertrophy and would provide further support that hCT1 can model the benefits of increased aerobic fitness (oxygen consumption rate) observed during endurance exercise training. It should also be noted that hCT1 results in increased expression of anti-oxidant regulatory factors (i.e., mitochondrial superoxide dismutase 2 and metallothionein 2A), a mechanism that is consistent with enhanced metabolic control (Supplementary information, Table S1). Finally, studies have shown that CT1 can significantly improve mitochondrial dysfunction leading to neuroprotection in a mouse model by increasing mitochondrial cytochrome oxidase activity and ATP energy levels, while decreasing reactive oxygen species production^43^, observations that are consistent with the data presented here.

In addition to elevated vascularity and metabolic profile, we assessed whether hCT1 limited fibrosis/fibrotic deposition, a phenotypic change that is consistent with pathologic hypertrophy^44^. We noted that hCT1-treated animals had no appreciable increase in fibrotic deposition compared with PE-treated animals, which displayed robust elevations in collagen deposits (Figure 4G-4J). hCT1-treated cardiac fibroblasts were also devoid of the robust increase in Galectin-3 that occurs during exposure to PE (Supplementary information, Figure S6A-S6C). Collectively, these data support the contention that CT1 induces multiple cellular adaptations to ensure a matched organ level response to maintain and augment cardiac function.

### hCT1 stimulation improves cardiac function in a model of severe right heart failure

The capacity for hCT1 to induce beneficial myocardial remodeling suggests that this protein may provide a tractable means to limit the progression of cardiac pathologies. To test this general premise, we examined whether hCT1 administration could limit and/or improve cardiac performance in a model of RHF due to severe pulmonary arterial hypertension (PAH)-induced inhibition of VEGFR2 with SU5416 (SU) followed by a 3-week exposure to hypoxia (Hx). The SUHx model faithfully reproduces many of the features of human PAH, which develops from a generalized thickening and obliteration of distal lung arterioles^45,46,47^. The severity of symptoms and the prognosis in PAH is largely driven by the degree to which the RV can adapt to the marked increases in afterload, and inability to compensate results in progressive RHF, reduced cardiac output, and ultimately death.

**Figure 5:**
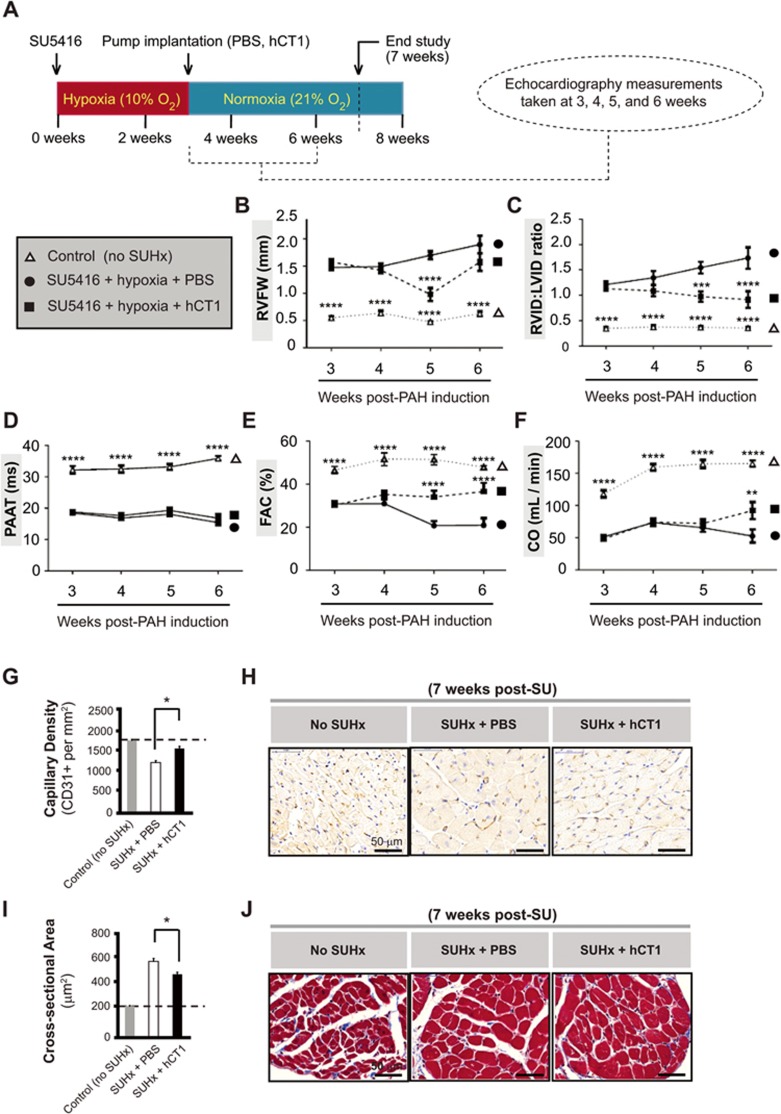
hCT1 attenuates the morphologic and hemodynamic effects of pulmonary arterial hypertension (PAH) in the Sugen/hypoxia (SUHx) rat model. **(A)** PAH was induced by administering a single subcutaneous injection of SU5416 (SU) at 20 mg/kg (VEGF receptor antagonist) followed by exposure to chronic hypoxia (Hx) at 10% oxygen for 3 weeks. Rats were recovered for 24 h in normoxia before subcutaneous implantation of osmotic minipumps containing hCT1 (6 μg/kg/h) or phosphate-buffered saline (PBS). Echocardiography was conducted and the following parameters were measured: right ventricle free wall thickness (RVFW; **B**), right to left ventricle internal diameter ratio (RVID:LVID; **C**), pulmonary artery acceleration time (PAAT; **D**), fractional area change (FAC; **E**), and cardiac output (CO; **F**). Control rats were not exposed to SU5416 or hypoxia. **(B-F)** Significant differences were observed between the PAH-induced SUHx rat groups (hCT1 or PBS) and the control rat group (*n* = 3; ^****^P < 0.0001). At 5-6 weeks, hCT1-treated SUHx animals showed a significant increase in CO **(F)** and FAC **(E)**, and a decrease in RVID:LVID **(C)** and RVFW **(B)** versus the PBS-treated SUHx group (*n* = 3; ^**^P < 0.01, ^***^P < 0.001 and ^****^P < 0.0001 ). **(G, H)** hCT1 treatment significantly increased capillary density versus PBS-treated SUHx animals (*n* = 3; ^*^P < 0.05). **(H)** Capillaries stained with endothelial-specific CD31+ antibody followed by chromogenic detection (DAB, brown). Nuclei counterstained with Hematoxylin. Scale bar, 50 μm. **(I, J)** hCT1 treatment significantly decreased cardiomyocyte cross-sectional area in the right ventricle versus PBS-treated SUHx animals (*n* = 3; ^*^P < 0.05). **(J)** Masson's Trichrome staining of heart cross-sectional area. Scale bar, 50 μm.

Our initial experiments revealed that systemic delivery of hCT1 protein during early stages of hypoxia exposure (prior to the development of RHF) provided an effective means to prevent the onset of RHF (Supplementary information, Figure S7A and S7B). However, this was associated with improvements in pulmonary hemodynamics, possibly due to the beneficial effects of the pro-angiogenic actions of hCT1 on the development of this disease. Therefore, we tested whether hCT1 protein delivery would mitigate RHF at later stages of disease in the SUHx model, when the severe PAH was fully established and RHF was already present. Here, we observed that delivery of hCT1 protein (beginning after 3 weeks of hypoxia exposure) was sufficient to reverse the RHF pathology, where hCT1-treated SUHx animals displayed significant improvement in both contractile function (cardiac output, fractional area change) and structural dimensions of the heart (RVID:LVID ratio) (Figure 5A-5C; 5E and 5F). In addition, hCT1-treated SUHx animals displayed increased RV vascularity (increased capillary density as measured by CD31 staining) and reduced RV cardiomyocyte cell size with a characteristic eccentric growth phenotype, when compared to sham-treated SUHx animals (Figure 5G-5J). Importantly, hCT1 treatment did not mitigate or lessen the elevated pulmonary pressures in the SUHx model (Figure 5D), strongly suggesting that hCT1 improved cardiac function by inducing heart/myocardial specific remodeling events.

## Discussion

Here, we demonstrate the capacity of a single exogenously delivered protein, CT1, to produce beneficial remodeling of the heart. The cardiac adaptations elicited by CT1 display the essential characteristics and benefits that are associated with endurance exercise adaptation, all of which are fully reversible when CT1 protein delivery is discontinued. Remarkably, administering CT1 alone achieves an organ level reorganization of the heart, impacting the myocardial and vascular cell components through a cardiomyocyte instructed response, i.e., CT1 stimulates both cell growth and a pro-vascular paracrine signal in the cardiomyocyte population.

Heart failure is a leading cause of morbidity and mortality in affluent societies and a growing epidemic worldwide^48^. The most intensely studied form of the disease, congestive HF, can be effectively managed with combinatorial interventions that enhance contractile performance, while reducing afterload on the weakened muscle (through diuretic-mediated reduction in fluid levels)^49^. RHF, which occurs when the right heart cannot adapt to an increase in afterload, has emerged as an equal if not greater public health crisis. This results from an increase in pulmonary arterial pressure or pulmonary hypertension (PH). Pulmonary arterial hypertension (PAH) is a rare but serious form of PH; however, by far the most common cause of PH is HF with preserved ejection fraction (HFpEF). Despite the important progress in the treatment of left HF with reduced ejection fraction, effective pharmacologic management of RHF, particularly in the context of HFpEF, has proven to be an intractable problem^50^. In addition, patients with RHF, regardless of etiology, have a very limited capacity to engage in and derive the myocardial benefits that are associated with aerobic exercise programs^50^. Our observations suggest that CT1 can dramatically improve cardiac performance in RHF, whereby the failing myocardium is functionally reprogrammed at the whole organ level.

**Figure 6:**
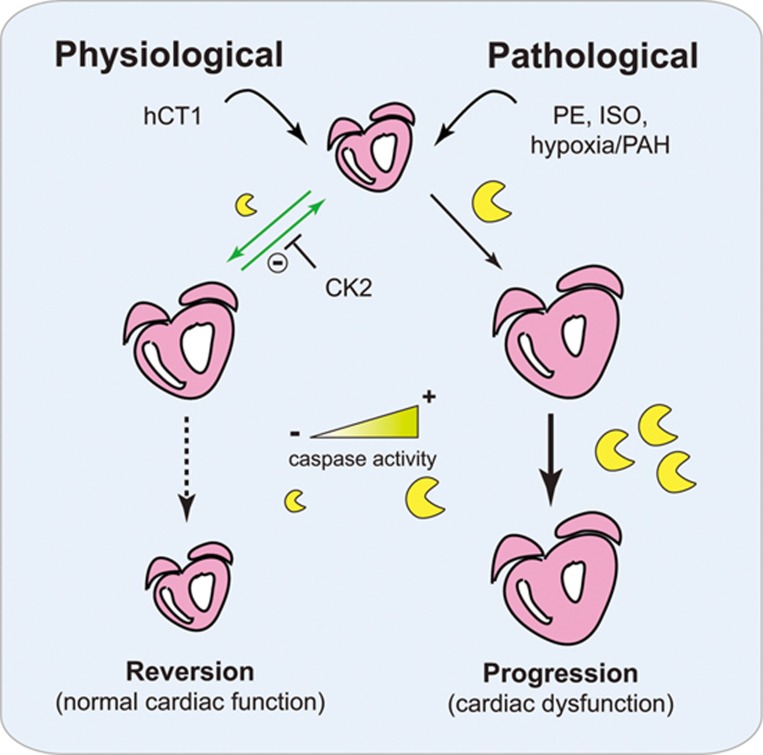
Proposed model of hCT1-mediated physiologic cardiac hypertrophy. hCT1 promotes reversible and beneficial cardiac remodeling by restraining caspase activation via CK2; whereas pathological stimulation (with PE, ISO, or hypoxia/PAH) causes unrestricted caspase activation with progression to cardiac dysfunction. Heart (pink), caspases (yellow), hCT1 (human cardiotrophin 1), PE (phenylephrine), ISO (isoproterenol), CK2 (casein kinase 2), PAH (pulmonary arterial hypertension).

The myocardial remodeling activity of CT1 is relayed through a complex and self-adjusting molecular signal cascade. For example, CT1 engages a temporally brief upregulation in the mitochondrial intrinsic cell death pathway, culminating in a pulse of caspase 3 activation. Robust activation of this signal cascade is a key feature of pathologic hypertrophy^33^, yet CT1 appears to deploy the cellular remodeling capacity of caspase 3 as a conduit to initiate physiologic growth. Our observations imply that caspase 3 stimulates an overlapping gene expression program during physiologic and pathologic hypertrophy, i.e., Mef2-dependent transcription, and that varying the duration of this biologic signal may be a primary mechanism to manage the divergent outcomes (Figure 6). CT1 activation of CK2 appears to be central to the process of restrained caspase signaling, as inhibition of this kinase leads to more robust caspase activity and a pathologic hypertrophy phenotype. How CT1 signals to CK2 is not entirely clear, although a post gp130-mediated cascade is a likely conduit. In the erythroleukemic cells, cytokine stimulation has been shown to promote a direct physical interaction between CK2 and the gp130 scaffold kinases JAK1/JAK2, resulting in an autoactivation loop between the JAKs and CK2^51^. Whether this regulatory network is operant in CT1-stimulated cardiomyocytes will require further investigation. Our experiments strongly suggest that CT1 stimulation of CK2 function is critical to limit caspase activation, a key molecular step in establishing the physiologic hypertrophy phenotype. CK2 is a holoenzyme that is constitutively active^36,37^, as such our data suggest it is most likely that CT1 stimulation limits the interaction between the kinase complex and caspase 3 and or the relevant caspase 3 substrates, rather than directly increasing CK2 activity. We have noted that during hypertrophy stimulation, the caspase 3 activation pattern is tightly scripted within the cell, forming discrete foci in peri-nuclear locations^36^. We attempted to monitor a CK2/caspase 3 interaction via proximity ligation assay (PLA), but were unable to establish an interaction between these enzymes using this methodology. The activated caspase 3 protein foci suggest that the protease may exist within aggregated structures, a condition that would not be conducive to monitoring protein interactions via PLA.

This variable signal hypothesis is consistently demonstrated in the developmental biology literature, where temporal activation of caspase 3 acts as a critical inductive cue for cell differentiation, while sustained activation leads to cell death within the same cell population^30,31^. Certainly, a temporal removal of Mef2 repression by caspase targeting of HDAC3 (as we have noted in^33^) would provide an amenable mechanism to titrate Mef2 activity, with a more robust activation of this pathway a characteristic of pathologic hypertrophy. Indeed, prior observations have shown that complete deletion of HDAC3 is synonymous with unrestrained pathologic myocardial growth^52^. Nevertheless, caspase 3 cleavage of HDAC3 is unlikely to solely account for the profound phenotypic variation between physiologic versus pathologic hypertrophy. Here, we favor the supposition that sustained caspase 3 activation leads to the targeting of a cohort of substrates that alter the structural integrity of the cardiomyocyte, and that these substrates are either inaccessible or subject to very limited proteolytic targeting when caspase 3 is transiently engaged by CT1. Indeed, caspase cleavage of contractile proteins such as Troponin T and myosin light chain have been noted in nonapoptotic cardiomyocytes during progression to dilation and failure^53,54^, consistent with the CT1-inspired limited caspase activation model we favor (Figure 6).

It is generally accepted that prolonged pathological cardiac hypertrophy frequently transitions to HF, which causes eccentric hypertrophy with chamber dilation and thinning of the myocardium^3^. Although studies have shown that increased cardiomyocyte lengthening contributes to chamber dilation and myocardial thinning in HF^55^, there is an appreciable number of studies that provide compelling evidence that exercise training also induces a beneficial cardiomyocyte response, in both morphology and function, by means of cardiomyocyte lengthening/elongation^56,57,58,59^. For example, low-intensity endurance training in rats resulted in eccentric hypertrophy of the left ventricle (LV) with increased length to width ratio of cardiomyocytes^57^. Although RV cardiomyocytes showed no change in length to width ratio, the authors postulate that the adaptive response of RV cardiomyocytes might depend on a more prolonged or intense exercise regimen such as high-intensity endurance training. Furthermore, cardiomyocyte lengthening was positively correlated with aerobic fitness in rats exposed to exercise treadmill training and detraining^59^. With training, cardiomyocyte length increased along with aerobic fitness. However, most of the aerobic fitness gained over 2-3 months was lost within 4 weeks of detraining, including regression of cardiomyocyte length back to sedentary control values^59^.

It is also important to note that programmed cell death (apoptosis) of cardiomyocytes is an essential process in the progression of dilated cardiomyopathy (DCM) and HF, and patients with DCM and HF have significantly higher apoptosis than control patients^60,61^. It is reasonable to conjecture that much of the eccentric hypertrophy and cardiomyocyte lengthening seen in the progression of DCM and HF is likely attributed to the indirect stretching of a weakened myocardium, which is a secondary effect to the direct loss of cardiomyocytes due to apoptosis. In the context of our study and our proposed model (Figure 6), we show that hCT1 stimulation does not lead to cell death, but rather, the observed cardiomyocyte lengthening is a result of specific engagement of a beneficial and reversible physiological hypertrophy process.

Moreover, there is plausible evolutionary evidence indicating that cellular elongation (or length to width ratio increase) provides a survival advantage to a cell or an organism^62^. Remarkably, some cells respond to stress (nutrient deprivation) by changing shapes via elongation (i.e., similar to eccentric growth and increased length to width ratio with hCT1 stimulation), which maximizes the “surface area to volume ratio” and this allows for efficient nutrient uptake during times of stress. On the contrary, spherical cells (i.e., similar to concentric growth and reduced length to width ratio with PE) have the worst possible shape for efficient nutrient/substrate uptake. This could partly account for the effects seen with pathological hypertrophy due to the inability of the hypertrophied cardiomyocyte to meet metabolic demands.

We have noted that CT1 engages signals that are proto-typical of the larger IL-6 cytokine family and are not conserved responses to pathologic hypertrophy agonists *per se*, i.e., gp130-mediated activation of STAT-dependent transcriptional activity. The STAT transcriptional response was sustained and likely contributes to the unique phenotypic outcome that characterizes physiologic hypertrophy and its beneficial remodeling events. At this stage we do not understand precisely how STAT-dependent signaling manifest change within the adapting cardiomyocyte. There is evidence to suggest that STAT activity can modify contractile protein content through upregulation of angiotensin gene expression, while promoting a pro-survival anti-apoptotic response^63^. This observation is certainly consistent with our studies, where CT1 stimulates a restricted caspase activation pattern. STAT transcriptional activity may also be a primary mechanism by which CT1 engages the cardiomyocyte production of angiogenic factors, i.e., STAT3 has been shown to be essential for promoting pro-angiogenic activity/capillarization within the myocardium^64,65^.

The myocardial and vascular remodeling capacity of CT1 implies that it may be effective in combating myopathies and ischemic heart disease in general. For example, the scalable and reversible hypertrophy that derives from CT1 stimulation may improve the contractile function of the post-infarct myocardium, while the pro-angiogenic and anti-fibrotic activities would curtail the loss in functional myocardium that occurs in the weeks following the initial ischemic damage. Indeed, preliminary experiments demonstrate that hCT1 protein can reduce scar size and improve contractile performance in a rodent infarct model up to 8 weeks from the initial insult (ligation of the left descending coronary artery; Supplementary information, Figure S7A and S7B). The rodent myocardium is more robust in the formation of collateral vessels compared to the human heart, nevertheless these observations provide a strong impetus to explore CT1 as a viable myocardial rejuvenation therapy in ischemic disease settings. Collectively, our studies suggest that CT1 protein therapy may provide a tractable means to improve cardiac function across a broad range of myopathic and ischemic disease.

Although pathologic hypertrophy frequently becomes irreversible and transitions to HF^66^, the phenotype can be reversed under limited circumstances. For example, inhibition of cGMP phosphodiesterase 5A (PDE5A) has been shown to prevent and reverse pressure overload-induced pathologic cardiac hypertrophy^67^. Also, calcineurin-dependent pathologic cardiac hypertrophy is partially reversible upon removal of calcineurin activity^68^. Furthermore, common anti-hypertensive treatments (e.g., β-blockers and angiotensin receptor blockers) can lead to partial reversion of pathologic hypertrophy in patients with hypertension^69^. Although these treatments reverse pathologic cardiac growth, the reversion is not complete as there is remnant fibrosis present. One of the most essential features distinguishing physiological from pathological hypertrophy is the capacity for complete growth reversion without residual fibrotic pathology^70^. As such, our observations are consistent with the hypothesis that hCT1 induces reversible physiological hypertrophy.

Finally, it is germane to note that therapeutic strategies designed to interdict or block caspase 3 activity, as a means to reduce myocardial cell death, are destined to fail. The transient activation of caspase 3 is a key step in the beneficial remodeling process afforded by CT1, and may also be central to exercise and pregnancy-associated myocardial adaptation. As such, indiscriminate disruption in the baseline activity of this protease will lead to a decline in adaptive capacity of the myocardium, while exacerbating disease progression.

## Materials and Methods

### Rats and mice

All animal studies (primary cell retrieval and *in vivo* analysis) were approved by the University of Ottawa Animal Care Committee. Sprague-Dawley rats, Fischer rats, and C57BL/6 mice were obtained from Charles River Laboratories (Wilmington, MA, USA).

### Neonatal rat primary cardiomyocytes

Primary neonatal rat cardiomyocytes were freshly isolated from hearts of 2-day-old Sprague-Dawley rats (Charles River Laboratories). Hearts were excised and placed in Joklik's-modified Eagle medium (JMEM). Ventricles were separated from the atria, minced with scalpel blades, and transferred to a 50 ml Falcon tube for digestion with 200 units per ml of Collagenase II (Worthington) in a total of 10 ml JMEM at 37 °C with gentle agitation. After 15 min, use a 25 ml pipet to slowly triturate (pipet up and down) the tissue and media about 2-3 times and then briefly wait for the tissue to settle at the bottom of the pipet. Then, pipet out (“spit”) the tissue into a clean tube for further Collagenase digestions while discarding the supernatant remaining in the pipet (“Digestion #0”), which contains mostly red blood cells and cell debris.

Perform another 4-5 rounds of Collagenase digests of the minced/digested heart tissue (as above); however, this time, the supernatant (containing an appreciable amount of primary cardiomyocytes) is transferred to a tube containing 10 ml of fetal bovine serum to stop the Collagenase reaction. This tube is centrifuged at 100× *g* for 5 min at room temperature to pellet the cells. The supernatant is discarded and the cells are resuspended in 1 ml Dulbecco's Modified Eagle Medium (DMEM) growth medium. Pool all the individual 1 ml cardiomyocyte fractions from the Collagenase digests into one tube. Transfer the volume of pooled cardiomyocytes onto a 70 μm filter pore size cell strainer (CellTreat) assembled on top of a 50 ml Falcon tube to help further remove cell clumps and cell debris. Wash the cell strainer with an additional 25 ml of DMEM growth medium.

Conduct a pre-plating step (to separate out cardiac fibroblasts and enrich for cardiomyocytes) by transferring the entire volume of flow-through onto a sterile 15 cm tissue culture dish and incubate at 37 °C for 30 min. Fibroblasts will adhere to the dish while cardiomyocytes remain in the media. Carefully collect the media using a 25 ml pipet and transfer to a new sterile 15 cm tissue culture dish for another round of pre-plating to further enrich for cardiomyocytes. After the second pre-plating incubation, transfer media to a sterile tube and centrifuge at 100× *g* for 5 min at room temperature. Discard the supernatant and resuspend the ventricular pellet in 5 ml of DMEM growth medium and this is the primary cardiomyocyte stock.

Count the number of cardiomyocytes using a Hemocytometer (cells per ml), then seed the desired number of cells directly onto collagen-coated tissue culture dishes and allow cells to recover overnight in DMEM growth medium in an incubator at 37 °C with 5% CO_2_. The next day, DMEM growth medium was replaced with serum-free (SF) medium and incubated for another day (up to 24 h) at 37 °C prior to conducting specific experiments.

### Adult mouse primary cardiomyocytes

Adult C57BL/6 mice (Charles River Laboratories) at 9 weeks of age were anesthetized using Avertin (0.4 mg/g) intraperitoneally. Thoracic cavity was then opened to expose the heart. Using a syringe pump, pre-warmed (37 °C) perfusion solution (126 mM NaCl, 4.4 mM KCl, 1 mM MgCl_2_, 4 mM NaHCO_3_, 30 mM 2,3-butanedione monoxime, 10 mM HEPES, 11 mM glucose, 0.5 mM EDTA, 0.09% Collagenase Type 2, 0.125% trypsin, 25 μM CaCl_2_ pH 7.4) was pumped through a 26G needle at a rate of 4 ml/min. The needle was inserted into the LV through the apex of the heart and the right atrium was cut. The mouse was then perfused for 5 min. The heart was then carefully removed and placed into a stop solution (126 mM NaCl, 4.4 mM KCl, 1 mM MgCl_2_, 4 mM NaHCO_3_, 30 mM 2,3-butanedione monoxime, 10 mM HEPES, 11 mM glucose, 0.5 mM EDTA, 100 μM CaCl_2_, 2% BSA pH 7.4) at 37 °C for 10 min. The heart was then cut into small pieces and gently triturated about 20-30 times using a wide-bore pipette in a petri dish. A 100 μm nylon mesh filter was used to remove large debris. Formalin (20%) was added in a 1:1 ratio to obtain a final concentration of 10% formalin. Cells were fixed for 15 min. The solution was then spun at 50× *g* for 5 min to pellet cardiomyocytes. Supernatant was disposed and cardiomyocytes were resuspended in PBS prior to conducting fluorescence cell staining.

### Recombinant hCT1 protein production

BL21 (DE3) cells (New England Biolabs) were transformed with a plasmid encoding wild-type hCT1. A fresh starter culture prepared from the transformed cells was diluted by 25-fold into 0.5 L Overnight Express autoinduction medium (EMD Millipore) prepared in 2×YT supplemented with 100 μg/ml kanamycin. The culture was grown in a shaking incubator at 37 °C for overnight. Cells were harvested and washed by PBS.

Cell pellet was resuspended in lysis buffer (50 mM Tris-HCl, pH 8.0, 100 mM NaCl, 1 mM EDTA, 10 mM DTT) supplemented with complete protease inhibitor cocktail (Roche) and 0.1% w/v sodium deoxycholate. Cells were lysed by sonication. The inclusion bodies contained in the pellet were washed three times by Triton wash buffer (50 mM Tris-HCl, 100 mM NaCl, 0.5% Triton X-100, 1 mM EDTA, and 1 mM DTT, pH 8.0) followed by three times by Tris wash buffer (50 mM Tris-HCl, 1 mM EDTA, and 1 mM DTT, pH 8.0).

Washed inclusion bodies were solubilized in solubilization buffer (100 mM sodium acetate, 2 M urea, 2 mM DTT, pH 3.5) at room temperature and centrifuged at 22 000× *g* for 30 min. The supernatant was dialyzed against Refolding buffer (20 mM HEPES, 2 mM DTT, pH 7.5) overnight at 4 °C . The refolded protein was centrifuged at 22 000× *g* for 30 min and filtered through a 0.22 μm filter and loaded to a HiTrap CM FF column (5 ml, GE Life Sciences) pre-equilibrated with solvent A (10 mM sodium phosphate, pH 7.5). Column was washed by 5 column volumes of solvent A, and the bound proteins were eluted stepwise by solvent A in 0.1 M NaCl then by solvent A in 1 M NaCl. The 0.1 M NaCl eluate was pooled and dialyzed against PBS overnight at 4 °C . Protein purity was estimated by SEC-HPLC to be > 95%. Mass spectrometry and Edman degradation indicated that the purified protein has a sequence of Ser2-Ala201 of the wild-type hCT1.

### Neonatal rat cardiomyocyte treatments

To induce hypertrophy, cardiomyocytes were treated for 24 (or 48) h with the following hypertrophic agonists diluted in SF control medium (SF, Ctrl): hCT1 recombinant protein at 0.5 nM (10 ng/ml) and PE at 100 μM. Serum-free control medium was also used as a non-hypertrophic control.

For caspase 9 inhibition, cardiomyocytes were pre-treated with N-benzyloxycarbonyl-Leu-Glu-His-Asp-fluoromethylketone (z-LEHD-fmk, 20 μM; BioVision) for 2 h before the induction of hypertrophy when fresh inhibitor was added. For effector caspase inhibition, cardiomyocytes were infected for 24 h with an AdV encoding the biologic effector caspase inhibitor p35 upstream of a GFP reporter (p35-AdV) prior to inducing hypertrophy with hCT1 or PE for a further 24 h. A GFP expressing reporter (GFP-AdV) was used as a control and both p35-AdV and GFP-AdV were infected at a mean of infectivity of 20 prior to hypertrophic stimulation. For Casein kinase 2 (CK2) inhibition, cardiomyocytes were pre-treated with 4,5,6,7-tetrabromobenzotriazole (TBBt, 50 μM; EMD Millipore) for 1 h before the induction of hypertrophy when fresh inhibitor was added.

To obtain conditioned medium for detection of the secreted angiogenic factor – VEGF, cardiomyocytes were seeded at a density of 1.0 × 10^6^ cells directly onto 60 mm diameter tissue culture dishes. Hypertrophy was induced with hCT1 (0.5 nM) or PE (100 μM) diluted in a total volume of 3 ml of control SF medium. After 24 h, the conditioned medium was transferred onto an Amicon Centrifugal Filter Device with a 3 kDa cut-off (EMD Millipore) and centrifuged at 4 000× *g* for 30 min at room temperature. The concentrated conditioned medium was analyzed by western immunoblotting. Recombinant VEGF protein (Abcam) was used as a positive control.

### Luciferase assays

Primary rat cardiomyocytes were seeded onto collagen-coated 48-well tissue culture plates (0.7 × 10^5^ cells/well) in DMEM growth medium. The following day, cells were transfected using Lipofectamine 2000 (Invitrogen), SF Opti-MEMα (Thermo Fisher), 4 ng of Renilla luciferase internal control plasmid, and 200 ng of Firefly luciferase reporter DNA plasmid under the control of: pro-hypertrophic gene promoters (NF-κB or Mef2), or a pro-survival gene promoter (Stat3), or a pro-inflammatory gene promoter (Stat1) for 5 h at 37 °C with 5% CO_2._ The medium was replaced with SF medium and cells were incubated overnight at 37 °C with 5% CO_2_. The next day, cardiomyocytes were treated with: non-hypertrophic SF medium control (Ctrl), hCT1 (0.5 nM), PE (100 μM), or procaspase 3-activating compound 1, PAC-1 (25 μM). Treatments were conducted at early time points (30 min, 1 h, and 3 h) and at a later time point (24 h). Luciferase activity (pro-hypertrophic reporter activation) was measured using the Dual Luciferase Assay System as per manufacturer's instructions (Promega). Luciferase values were normalized to the Renilla internal control values and to the SF medium control values.

### Oxygen consumption rate assay

Primary rat cardiomyocytes were seeded onto collagen-coated 98-well tissue culture plates (0.9 × 10^5^ cells/well) in DMEM growth medium. The next day, cell culture medium was replaced with SF medium for 24 h. Cardiomyocytes were treated for 1 or 24 h with the following hypertrophic agonists diluted in SF control medium (SF, Ctrl): hCT1 recombinant protein at 0.5 nM (10 ng/ml) and PE at 100 μM. Serum-free control medium was used as a non-hypertrophic control. The MitoXpress – Xtra Oxygen Consumption Rate (OCR) Assay kit was used to measure oxygen consumption according to manufacturer's instructions (Cayman Chemical). Glucose Oxidase enzyme was used as a positive control. The phosphorescent oxygen probe (MitoXpress – Xtra) was used to measure the rate of oxygen consumption in whole cells by standard fluorescence intensity analysis using an excitation of 380 nm and emission of 650 nm.

### Immunocytochemistry

Fluorescent cell staining was conducted on primary rat cardiomyocytes (or cardiac fibroblasts) that were seeded at a density of 2.7 × 10^5^ cells directly onto 25 mm diameter collagen-coated glass coverslips. Cells were washed three times in PBS (3 min each) followed by fixation in 4% paraformaldehyde for 30 min. Cells were then permeabilized in Blocking buffer (1% bovine serum albumin – BSA, 2% normal goat serum, 0.4% Tx-100 in PBS) for 15 min. Cells were then incubated for 2 h at room temperature with primary antibody (diluted in Blocking buffer) followed by three successive washes in PBS at 3 min each. Cells were then blocked again in Blocking buffer for 15 min followed by a 1 h incubation in fluorescently conjugated secondary antibody (diluted in Blocking buffer). Cells were then washed again in PBS (five times at 5 min each). Nuclei were counterstained with 4′,6-diamidino-2-phenylindole (DAPI) and cells were mounted onto microscope slides with DakoCytomation fluorescent mounting medium (Dako). Digital fluorescent images were obtained using the Zeiss AxioVision SE64 Imaging software (Zeiss).

Primary antibodies used: mouse anti-α-actinin (Sigma), rabbit anti-atrial natriuretic peptide (ANP; Abcam), rabbit anti-active caspase 9 (Abcam), rabbit anti-VEGF (Abcam), rabbit anti-vimentin (Abcam), mouse anti-Galectin-3 (Abcam).

Secondary antibodies used: goat anti-mouse-Cy3 and donkey anti-rabbit-Cy3 (EMD Millipore), Alexa Fluor-488 goat anti-rabbit and Alexa Fluor-647 donkey anti-mouse (ThermoFisher).

Fluorescent cell staining was also conducted on isolated adult primary mouse cardiomyocytes; however, a high-affinity fluorescently labeled phallotoxin probe was used to detect filamentous F-actin: Alexa Fluor-488 conjugated Phalloidin (ThermoFisher). Nuclei were counterstained with DAPI. Digital images were acquired by fluorescence microscopy and cells were analyzed using ImageJ.

### Western blotting

Cells were washed in PBS and harvested by centrifugation. Cells were then resuspended in lysis buffer supplemented with protease inhibitors (50 mM HEPES pH 7.5, 150 mM NaCl, 10% glycerol, 1% Tx-100, 1 mM EGTA, 1.5 mM MgCl_2_, 20 mM NaF, 10 mM sodium pyrophosphate, 0.3 mM PMSF, 2.5 mM sodium orthovanadate) and incubated at 4 °C with gentle rotation for 1 h. Crude lysate was centrifuged at 17 000× *g* for 10 min. Equal amounts (20 μg) of protein were separated by SDS-PAGE and transferred to PVDF membrane for immunoblotting. After blocking the membrane with TBST-milk (10 mM Tris pH 7.4, 150 mM NaCl, 0.05% Tween-20, 5% non-fat powdered milk), membranes were incubated with primary antibody overnight at 4 °C followed by incubation with horseradish peroxidase (HRP)-conjugated secondary antibody for 1 h. Protein expression was detected using the ECL detection kit (GE Healthcare).

Primary antibodies used: rabbit anti-active caspase 3 (Cell Signaling), rabbit anti-glyceraldehyde-3-phosphate dehydrogenase (GAPDH; Cell Signaling), rabbit anti-VEGF (Abcam).

Secondary antibodies used: goat anti-mouse IgG-HRP and goat anti-rabbit IgG-HRP (Bio-Rad).

### *In vivo* analysis

**Reversion study and 2-week-only analysis** (i) Preparation and surgical implantation of osmotic minipumps. Osmotic minipumps (Model 2002, Alzet; Durect) were used to infuse the following over 2 weeks: hCT1, PE, ISO, or PBS as a non-hypertrophic control. Alzet minipumps (Model 2002) have a reservoir volume of 200 μl and infuse at a steady rate of 0.5 μl/h for 2 weeks. hCT1 was diluted accordingly in PBS based on the average rat weight per group to give a final infusion dose of 6 μg/kg/h. Similarly, the infusion doses for PE and ISO were 10 and 1 mg/kg/d; respectively. All prepared minipumps (PBS, hCT1, PE, and ISO) were pre-equilibrated in normal saline (0.9% NaCl) overnight at 37 °C in order to reach a steady-state pumping rate of 0.5 μl/h at the time of subcutaneous implantation into Sprague-Dawley rats. Rats were anesthetized with a mixture of 2% Isoflurane and pure oxygen (at 1L per min). Under sterile conditions, a subcutaneous incision was made between the shoulder blades in the upper back using a scalpel blade and an osmotic minipump was gently inserted under the skin. The incision area was then firmly stapled and rats were placed back into their respective cages to recover.

(ii) For the Reversion study, osmotic minipumps (Model 2002) were implanted into three groups of rats. PBS control, hCT1 at 6 μg/kg/h, or PE at 10 mg/kg/d. Rats weighed an average of 300 g at time of surgery. After 2 weeks, cardiac echocardiography was performed, minipumps were then surgically removed, and rats were allowed to recover. At 6 weeks post withdrawal (i.e., at 8 weeks of the study), rats were subjected to another round of cardiac echocardiography and rats were killed and heart tissues were excised for analysis. Of note, the 2-week-only rat study group was divided into the following three groups: PBS control, hCT1 at 6 μg/kg/h, or ISO at 1 mg/kg/d.

(iii) Tissue analysis and histology. All excised hearts were placed in 10% formalin for at least 48-72 h prior to embedding in paraffin and sectioning. The following immunohistochemical analyses were conducted on sectioned tissues: hematoxylin and eosin (H&E) for general morphology, Masson's Trichrome for collagen/fibrotic deposition, and CD31+ (platelet endothelial cell adhesion molecule) staining for angiogenesis and capillary density. Analysis for CD31+ was antibody-based and utilized the colorimetric detection of a 3′,3′-Diaminobenzidine (DAB) chromogen (brown precipitate) according to manufacturer's instructions (MACH4 Detection System; BioCare Medical). Immunohistologic analyses were also conducted by the Pathology and Laboratory Medicine (PALM) Histology Core Facility at the University of Ottawa, Canada.

**Sugen/hypoxia (SUHx) rat analysis** (i) SUHx study outline. PAH was induced by administering a single subcutaneous injection of SU5416 (SU) at 20 mg/kg (a potent VEGFR – VEGFR antagonist) into Fischer rats followed by exposure to chronic hypoxia (CH; 10% oxygen) for 3 weeks. At 3 weeks, rats were recovered for 24 h under normoxic conditions (21% oxygen) before subcutaneous implantation of Osmotic minipumps (Model 2ML4) containing either hCT1 (6 μg/kg/h) or PBS. Cardiac echocardiography analysis was conducted weekly up to the end of study at 7 weeks at which time rats were killed and heart tissues excised for analysis and histology.

(ii) Preparation of osmotic minipumps for SUH analysis. After 3 weeks induction of PAH in Fischer rats followed by 24 h of recovery under normoxic conditions, osmotic minipumps (Model 2ML4, Alzet; Durect) were used to infuse hCT1. PBS was infused for the control rat group. Osmotic minipumps (Model 2ML4) have a reservoir volume of 2 ml and infuse at a steady rate of 2.5 μl/h for 4 weeks. hCT1 was diluted accordingly in PBS based on the average rat weight per group (150 g) to give a final infusion dose of 6 μg/kg/h. All prepared minipumps (PBS and hCT1) were pre-equilibrated in normal saline (0.9% NaCl) overnight at 37 °C in order to reach a steady-state pumping rate of 2.5 μl/h at the time of subcutaneous implantation.

**Adult mouse primary cardiomyocyte morphology analysis** (i) Study outline. Adult C57BL/6 mice (9 weeks of age) were subcutaneously implanted with Osmotic minipumps (Model 1002) containing: hCT1, PE, or PBS as a non-hypertrophic control. After 2 weeks of infusion, the mice were anesthetized and adult mouse primary cardiomyocytes were isolated by perfusing and digesting whole hearts (see protocol for isolating adult mouse cardiomyocytes). Cardiomyocytes were then stained using immunocytochemistry and analyzed for morphological changes.

(ii) Preparation and surgical implantation of osmotic minipumps. Osmotic minipumps (Model 1002, Alzet; Durect) were used to infuse the following over 2 weeks: PBS (control), hCT1 (6 μg/kg/h), and PE (10 mg/kg/d). Alzet minipumps (Model 1002) have a reservoir volume of 100 μl and infuse at a steady rate of 0.25 μl/h for 2 weeks All prepared minipumps (PBS, hCT1, and PE) were pre-equilibrated in normal saline (0.9% NaCl) overnight at 37 °C in order to reach a steady-state pumping rate of 0.25 μl/h at the time of subcutaneous implantation.

**Myocardial infarction analysis** Ligation of the left anterior descending coronary artery (LAD) was used to induce a myocardial infarct in rats. Male Sprague-Dawley rats weighing 250 g at the time of surgery were subjected to LAD ligation surgery or were sham operated (open cavity, no LAD). Following ligation, animals were given an intracardiac injection of hCT1-AdV at 7.5 × 10^5^ infectious particles per gram weight, administered into the middle region of the LV. A separate group of animals were given saline following LAD surgery. The animals were allowed to recover for 2 weeks followed by a second intracardiac booster injection of either hCT1-AdV or saline (as above).

At 8 weeks post myocardial infarction, LV function was measured for each animal using a 2 F high-fidelity micro-manometer catheter (Millar Institute) inserted into the LV through the right carotid artery. A Harvard Data Acquisition system (Harvard Apparatus) was used to acquire data and processed using AcqKnowledge III software (ACQ 3.2) for measurement of left ventricular end-diastolic pressure, a critical hemodynamic measurement of ventricular performance. Immediately after acquisition, animals were injected with a 2 M KCl solution to stop the heart in diastole. The heart was excised and fixed in 10% formalin prior to paraffin embedding. Approximately 5-8 μm sections were stained with H&E and counterstained with Masson's Trichrome to visualize the infarct area.

### Microarray analysis

Primary neonatal rat cardiomyocytes (previously seeded at 2.5 × 10^6^ onto collagen-coated 10 cm tissue culture dishes) were treated for 24 h with hCT1 (0.5 nM) or with control SF medium. Total RNA was isolated using a TRIzol reagent extraction method. Briefly, 2 ml of TRIzol (ThermoFisher) was added per dish and gently agitated for 5 min at room temperature to lyse cells. The lysate was transferred to a 15 ml Falcon tube and 0.4 ml of chloroform was added. The tube was vortexed for 20 s, left to rest/settle for 3 min, and centrifuged at 3 200× *g* for 25 min, all at room temperature. The clear upper aqueous layer (containing RNA) was transferred to a sterile eppendorf tube and an equal volume of isopropanol was added to precipitate RNA overnight at −20 °C. The RNA pellet was centrifuged at 10 000× *g* for 15 min at 4 °C, washed in 70% ethanol (RNase-free), and resuspended in 100 μl of sterile water (RNase-free).

The RNA was cleaned with the RNeasy MinElute Cleanup kit (Qiagen) and 300 ng of RNA was processed for microarray using the Affymetrix GeneChip WT PLUS Reagent Kit (Affymetrix) according to manufacturer's instructions. The microarray processing and bioinformatics analysis was conducted at the StemCore Facility of The Ottawa Hospital Research Institute, Canada. Raw data files for the microarray analysis have been deposited in the NCBI Gene Expression Omnibus under accession number GSE87521.

### Statistical analysis

All data are expressed as mean ± SEM. Statistical significance for multiple-group comparisons was determined using one-way ANOVA followed by post-hoc statistical analysis (GraphPad Prism software). Dunnett's test was used for pairwise comparisons of treatment groups with control groups and Tukey's test was used for pairwise comparisons between treatment groups. *P* < 0.05 was considered significant.

## Author Contributions

MA-G, CS, BJ, YD, JJW, CP, SB, PF, TTL, PF, FHHL, PGB, DJS, and LAM designed research; MA-G, CS, BJ, YD, JJW, CP, SB, PF, TTL, PF and PGB performed research; MA-G, CS, BJ, YD, JJW, CP, SB, PF, TTL, PF, FHHL, PGB, DJS, and LAM analyzed data; and MA-G, CS, DJS, and LAM wrote the paper.

## Competing Financial Interests

The authors declare no competing financial interests.

## Supplementary Information

Supplementary information, Figure S1 hCT1 engages a restricted activation of the intrinsic caspase-mediated cell death pathway.

Supplementary information, Figure S2 Pro-hypertrophic stimulation with hCT1 causes significant Stat3 activation.

Supplementary information, Figure S3 Casein kinase 2 (CK2) activation is required for hCT1 induced cardiomyocyte hypertrophy.

Supplementary information, Figure S4 VEGF expression induced by hCT1 stimulation is inhibited upon blocking caspase activity.

Supplementary information, Figure S5 hCT1 stimulation increases the oxygen consumption rate of cardiomyocytes.

Supplementary information, Figure S6 hCT1 stimulation of cardiac fibroblasts does not significantly induce expression of the pro-fibrotic marker Galectin-3.

Supplementary information, Figure S7 hCT1 attenuates the effects observed upon inducing myocardial infarction (MI) by ligation of the Left Anterior Descending (LAD) coronary artery.

Supplementary information, Table S1 Microarray analysis of hCT1 stimulated primary cardiomyocytes.

## References

[bib1] Hill JA, Olson EN. Cardiac plasticity. N Engl J Med 2008; 358:1370–1380. 18367740 10.1056/NEJMra072139

[bib2] Harvey PA, Leinwand LA. The cell biology of disease: cellular mechanisms of cardiomyopathy. J Cell Biol 2011; 194:355–365. 21825071 10.1083/jcb.201101100 PMC3153638

[bib3] van Berlo JH, Maillet M, Molkentin JD. Signaling effectors underlying pathologic growth and remodeling of the heart. J Clin Invest 2013; 123:37–45. 23281408 10.1172/JCI62839 PMC3533272

[bib4] Chung E, Leinwand LA. Pregnancy as a cardiac stress model. Cardiovasc Res 2014; 101:561–570. 24448313 10.1093/cvr/cvu013 PMC3941597

[bib5] Roh J, Rhee J, Chaudhari V, Rosenzweig A. The role of exercise in cardiac aging: from physiology to molecular mechanisms. Circ Res 2016; 118:279–295. 26838314 10.1161/CIRCRESAHA.115.305250 PMC4914047

[bib6] Bueno OF, De Windt LJ, Tymitz KM, et al. The MEK1-ERK1/2 signaling pathway promotes compensated cardiac hypertrophy in transgenic mice. EMBO J 2000; 19:6341–6350. 11101507 10.1093/emboj/19.23.6341 PMC305855

[bib7] Condorelli G, Drusco A, Stassi G, et al. Akt induces enhanced myocardial contractility and cell size *in vivo* in transgenic mice. Proc Natl Acad Sci USA 2002; 99:12333–12338. 12237475 10.1073/pnas.172376399 PMC129445

[bib8] Maillet M, van Berlo JH, Molkentin JD. Molecular basis of physiological heart growth: fundamental concepts and new players. Nat Rev Mol Cell Biol 2013; 14:38–48. 23258295 10.1038/nrm3495 PMC4416212

[bib9] Shiojima I, Walsh K. Regulation of cardiac growth and coronary angiogenesis by the Akt/PKB signaling pathway. Genes Dev 2006; 20:3347–3365. 17182864 10.1101/gad.1492806

[bib10] Shiojima I, Yefremashvili M, Luo Z, et al. Akt signaling mediates postnatal heart growth in response to insulin and nutritional status. J Biol Chem 2002; 277:37670–37677. 12163490 10.1074/jbc.M204572200

[bib11] Duerr RL, Huang S, Miraliakbar HR, Clark R, Chien KR, Ross J. Jr. Insulin-like growth factor-1 enhances ventricular hypertrophy and function during the onset of experimental cardiac failure. J Clin Invest 1995; 95:619–627. 7860746 10.1172/JCI117706 PMC295527

[bib12] McMullen JR, Shioi T, Huang WY, et al. The insulin-like growth factor 1 receptor induces physiological heart growth via the phosphoinositide 3-kinase(p110alpha) pathway. J Biol Chem 2004; 279:4782–4793. 14597618 10.1074/jbc.M310405200

[bib13] Sapra G, Tham YK, Cemerlang N, et al. The small-molecule BGP-15 protects against heart failure and atrial fibrillation in mice. Nat Commun 2014; 5:5705. 25489988 10.1038/ncomms6705

[bib14] Pennica D, King KL, Shaw KJ, et al. Expression cloning of cardiotrophin 1, a cytokine that induces cardiac myocyte hypertrophy. Proc Natl Acad Sci USA 1995; 92:1142–1146. 7862649 10.1073/pnas.92.4.1142 PMC42654

[bib15] Wollert KC, Taga T, Saito M, et al. Cardiotrophin-1 activates a distinct form of cardiac muscle cell hypertrophy. Assembly of sarcomeric units in series VIA gp130/leukemia inhibitory factor receptor-dependent pathways. J Biol Chem 1996; 271:9535–9545. 8621626 10.1074/jbc.271.16.9535

[bib16] Ishikawa M, Saito Y, Miyamoto Y, et al. A heart-specific increase in cardiotrophin-1 gene expression precedes the establishment of ventricular hypertrophy in genetically hypertensive rats. J Hypertens 1999; 17:807–816. 10459879 10.1097/00004872-199917060-00013

[bib17] Jin H, Yang R, Keller GA, et al. *In vivo* effects of cardiotrophin-1. Cytokine 1996; 8:920–926. 9050750 10.1006/cyto.1996.0123

[bib18] Lopez B, Gonzalez A, Querejeta R, Larman M, Rabago G, Diez J. Association of cardiotrophin-1 with myocardial fibrosis in hypertensive patients with heart failure. Hypertension 2014; 63:483–489. 24366078 10.1161/HYPERTENSIONAHA.113.02654

[bib19] Schillaci G, Pucci G, Perlini S. From hypertension to hypertrophy to heart failure: the role of cardiotrophin-1. J Hypertens 2013; 31:474–476. 23615209 10.1097/HJH.0b013e32835ed4bb

[bib20] Fischer P, Hilfiker-Kleiner D. Survival pathways in hypertrophy and heart failure: the gp130-STAT axis. Basic Res Cardiol 2007; 102:393–411. 17918316 10.1007/s00395-007-0674-z

[bib21] Hirota H, Chen J, Betz UA, et al. Loss of a gp130 cardiac muscle cell survival pathway is a critical event in the onset of heart failure during biomechanical stress. Cell 1999; 97:189–198. 10219240 10.1016/s0092-8674(00)80729-1

[bib22] Yoshida K, Taga T, Saito M, et al. Targeted disruption of gp130, a common signal transducer for the interleukin 6 family of cytokines, leads to myocardial and hematological disorders. Proc Natl Acad Sci USA 1996; 93:407–411. 8552649 10.1073/pnas.93.1.407 PMC40247

[bib23] Calabro P, Limongelli G, Riegler L, et al. Novel insights into the role of cardiotrophin-1 in cardiovascular diseases. J Mol Cell Cardiol 2009; 46:142–148. 19059413 10.1016/j.yjmcc.2008.11.002

[bib24] Aguilar-Melero P, Luque A, Machuca MM, et al. Cardiotrophin-1 reduces ischemia/reperfusion injury during liver transplant. J Surg Res 2013; 181:e83–91. 22906559 10.1016/j.jss.2012.07.046

[bib25] Iniguez M, Berasain C, Martinez-Anso E, et al. Cardiotrophin-1 defends the liver against ischemia-reperfusion injury and mediates the protective effect of ischemic preconditioning. J Exp Med 2006; 203:2809–2815. 17178916 10.1084/jem.20061421 PMC2118168

[bib26] Moreno-Aliaga MJ, Perez-Echarri N, Marcos-Gomez B, et al. Cardiotrophin-1 is a key regulator of glucose and lipid metabolism. Cell Metab 2011; 14:242–253. 21803294 10.1016/j.cmet.2011.05.013

[bib27] Kolodziejczyk SM, Wang L, Balazsi K, DeRepentigny Y, Kothary R, Megeney LA. MEF2 is upregulated during cardiac hypertrophy and is required for normal post-natal growth of the myocardium. Curr Biol 1999; 9:1203–1206. 10531040 10.1016/S0960-9822(00)80027-5

[bib28] Naya FJ, Black BL, Wu H, et al. Mitochondrial deficiency and cardiac sudden death in mice lacking the MEF2A transcription factor. Nat Med 2002; 8:1303–1309. 12379849 10.1038/nm789

[bib29] van Rooij E, Fielitz J, Sutherland LB, et al. Myocyte enhancer factor 2 and class II histone deacetylases control a gender-specific pathway of cardioprotection mediated by the estrogen receptor. Circ Res 2010; 106:155–165. 19893013 10.1161/CIRCRESAHA.109.207084 PMC3623688

[bib30] Abdul-Ghani M, Megeney LA. Rehabilitation of a contract killer: caspase-3 directs stem cell differentiation. Cell Stem Cell 2008; 2:515–516. 18522841 10.1016/j.stem.2008.05.013

[bib31] Dick SA, Megeney LA. Cell death proteins: an evolutionary role in cellular adaptation before the advent of apoptosis. Bioessays 2013; 35:974–983. 23943356 10.1002/bies.201300052

[bib32] Fuchs Y, Steller H. Programmed cell death in animal development and disease. Cell 2011; 147:742–758. 22078876 10.1016/j.cell.2011.10.033 PMC4511103

[bib33] Putinski C, Abdul-Ghani M, Stiles R, et al. Intrinsic-mediated caspase activation is essential for cardiomyocyte hypertrophy. Proc Natl Acad Sci USA 2013; 110:E4079–4087. 24101493 10.1073/pnas.1315587110 PMC3808644

[bib34] Regis G, Pensa S, Boselli D, Novelli F, Poli V. Ups and downs: the STAT1:STAT3 seesaw of interferon and gp130 receptor signalling. Semin Cell Dev Biol 2008; 19:351–359. 18620071 10.1016/j.semcdb.2008.06.004

[bib35] de Almeida JC, Alves CL, de Abreu LC, et al. Involvement of the atrial natriuretic peptide in cardiovascular pathophysiology and its relationship with exercise. Int Arch Med 2012; 5:4. 22313592 10.1186/1755-7682-5-4 PMC3395876

[bib36] Dick SA, Chang NC, Dumont NA, et al. Caspase 3 cleavage of Pax7 inhibits self-renewal of satellite cells. Proc Natl Acad Sci USA 2015; 112:E5246–5252. 26372956 10.1073/pnas.1512869112 PMC4586827

[bib37] Duncan JS, Turowec JP, Duncan KE, et al. A peptide-based target screen implicates the protein kinase CK2 in the global regulation of caspase signaling. Sci Signal 2011; 4:ra30. 21558555 10.1126/scisignal.2001682

[bib38] Hudlicka O, Brown M, Egginton S. Angiogenesis in skeletal and cardiac muscle. Physiol Rev 1992; 72:369–417. 1372998 10.1152/physrev.1992.72.2.369

[bib39] Giordano FJ, Gerber HP, Williams SP, et al. A cardiac myocyte vascular endothelial growth factor paracrine pathway is required to maintain cardiac function. Proc Natl Acad Sci USA 2001; 98:5780–5785. 11331753 10.1073/pnas.091415198 PMC33290

[bib40] Osugi T, Oshima Y, Fujio Y, et al. Cardiac-specific activation of signal transducer and activator of transcription 3 promotes vascular formation in the heart. J Biol Chem 2002; 277:6676–6681. 11744720 10.1074/jbc.M108246200

[bib41] Zhou Y, Bourcy K, Kang YJ. Copper-induced regression of cardiomyocyte hypertrophy is associated with enhanced vascular endothelial growth factor receptor-1 signalling pathway. Cardiovasc Res 2009; 84:54–63. 19542178 10.1093/cvr/cvp178 PMC2741345

[bib42] Abel ED, Doenst T. Mitochondrial adaptations to physiological vs. pathological cardiac hypertrophy. Cardiovasc Res 2011; 90:234–242. 21257612 10.1093/cvr/cvr015 PMC3115280

[bib43] Wang D, Liu X, Liu Y, Shen G, Zhu X, Li S. Treatment effects of Cardiotrophin-1 (CT-1) on streptozotocin-induced memory deficits in mice. Exp Gerontol 2017; 92:42–45. 28285145 10.1016/j.exger.2017.03.007

[bib44] Piek A, de Boer RA, Sillje HH. The fibrosis-cell death axis in heart failure. Heart Fail Rev 2016; 21:199–211. 26883434 10.1007/s10741-016-9536-9 PMC4762920

[bib45] Abe K, Toba M, Alzoubi A, et al. Formation of plexiform lesions in experimental severe pulmonary arterial hypertension. Circulation 2010; 121:2747–2754. 20547927 10.1161/CIRCULATIONAHA.109.927681

[bib46] Jiang B, Deng Y, Suen C, et al. Marked strain-specific differences in the SU5416 rat model of severe pulmonary arterial hypertension. Am J Respir Cell Mol Biol 2016; 54:461–468. 26291195 10.1165/rcmb.2014-0488OC

[bib47] Ryan JJ, Marsboom G, Fang YH, et al. PGC1alpha-mediated mitofusin-2 deficiency in female rats and humans with pulmonary arterial hypertension. Am J Respir Crit Care Med 2013; 187:865–878. 23449689 10.1164/rccm.201209-1687OC PMC3707374

[bib48] Ziaeian B, Fonarow GC. Epidemiology and aetiology of heart failure. Nat Rev Cardiol 2016; 13:368–378. 26935038 10.1038/nrcardio.2016.25 PMC4868779

[bib49] Yancy CW, Jessup M, Bozkurt B, et al. 2013 ACCF/AHA guideline for the management of heart failure: a report of the American College of Cardiology Foundation/American Heart Association Task Force on Practice Guidelines. J Am Coll Cardiol 2013; 62:e147–239. 23747642 10.1016/j.jacc.2013.05.019

[bib50] Shah SJ, Kitzman DW, Borlaug BA, et al. Phenotype-specific treatment of heart failure with preserved ejection fraction: a multiorgan roadmap. Circulation 2016; 134:73–90. 27358439 10.1161/CIRCULATIONAHA.116.021884 PMC4930115

[bib51] Zheng Y, Qin H, Frank SJ, et al. A CK2-dependent mechanism for activation of the JAK-STAT signaling pathway. Blood 2011; 118:156–166. 21527517 10.1182/blood-2010-01-266320 PMC3139382

[bib52] Montgomery RL, Potthoff MJ, Haberland M, et al. Maintenance of cardiac energy metabolism by histone deacetylase 3 in mice. J Clin Invest 2008; 118:3588–3597. 18830415 10.1172/JCI35847 PMC2556240

[bib53] Communal C, Sumandea M, de Tombe P, Narula J, Solaro RJ, Hajjar RJ. Functional consequences of caspase activation in cardiac myocytes. Proc Natl Acad Sci USA 2002; 99:6252–6256. 11972044 10.1073/pnas.092022999 PMC122935

[bib54] Moretti A, Weig HJ, Ott T, et al. Essential myosin light chain as a target for caspase-3 in failing myocardium. Proc Natl Acad Sci USA 2002; 99:11860–11865. 12186978 10.1073/pnas.182373099 PMC129359

[bib55] Gerdes AM, Kellerman SE, Moore JA, et al. Structural remodeling of cardiac myocytes in patients with ischemic cardiomyopathy. Circulation 1992; 86:426–430. 1638711 10.1161/01.cir.86.2.426

[bib56] Carneiro-Junior MA, Peluzio MC, Silva CH, et al. Exercise training and detraining modify the morphological and mechanical properties of single cardiac myocytes obtained from spontaneously hypertensive rats. Braz J Med Biol Res 2010; 43:1042–1046. 21049244 10.1590/s0100-879x2010007500117

[bib57] Carneiro-Junior MA, Primola-Gomes TN, Quintao-Junior JF, et al. Regional effects of low-intensity endurance training on structural and mechanical properties of rat ventricular myocytes. J Appl Physiol 2013; 115:107–115. 23640594 10.1152/japplphysiol.00041.2013

[bib58] Carneiro-Junior MA, Quintao-Junior JF, Drummond LR, et al. The benefits of endurance training in cardiomyocyte function in hypertensive rats are reversed within four weeks of detraining. J Mol Cell Cardiol 2013; 57:119–128. 23376037 10.1016/j.yjmcc.2013.01.013

[bib59] Kemi OJ, Haram PM, Wisloff U, Ellingsen O. Aerobic fitness is associated with cardiomyocyte contractile capacity and endothelial function in exercise training and detraining. Circulation 2004; 109:2897–2904. 15173028 10.1161/01.CIR.0000129308.04757.72

[bib60] Glumac S, Pejic S, Kostadinovic S, Stojsic Z, Vasiljevic J. Apoptosis in endomyocardial biopsies from patients with dilated cardiomyopathy. Folia Biol 2016; 62:207–211. 10.14712/fb2016062050207 27978416

[bib61] van Empel VP, Bertrand AT, Hofstra L, Crijns HJ, Doevendans PA, De Windt LJ. Myocyte apoptosis in heart failure. Cardiovasc Res 2005; 67:21–29. 15896727 10.1016/j.cardiores.2005.04.012

[bib62] Young KD. The selective value of bacterial shape. Microbiol Mol Biol Rev 2006; 70:660–703. 16959965 10.1128/MMBR.00001-06 PMC1594593

[bib63] Kunisada K, Negoro S, Tone E, et al. Signal transducer and activator of transcription 3 in the heart transduces not only a hypertrophic signal but a protective signal against doxorubicin-induced cardiomyopathy. Proc Natl Acad Sci USA 2000; 97:315–319. 10618415 10.1073/pnas.97.1.315 PMC26660

[bib64] Hilfiker-Kleiner D, Hilfiker A, Fuchs M, et al. Signal transducer and activator of transcription 3 is required for myocardial capillary growth, control of interstitial matrix deposition, and heart protection from ischemic injury. Circ Res 2004; 95:187–195. 15192020 10.1161/01.RES.0000134921.50377.61

[bib65] Obana M, Maeda M, Takeda K, et al. Therapeutic activation of signal transducer and activator of transcription 3 by interleukin-11 ameliorates cardiac fibrosis after myocardial infarction. Circulation 2010; 121:684–691. 20100971 10.1161/CIRCULATIONAHA.109.893677

[bib66] Bernardo BC, Weeks KL, Pretorius L, McMullen JR. Molecular distinction between physiological and pathological cardiac hypertrophy: experimental findings and therapeutic strategies. Pharmacol Ther 2010; 128:191–227. 20438756 10.1016/j.pharmthera.2010.04.005

[bib67] Takimoto E, Champion HC, Li M, et al. Chronic inhibition of cyclic GMP phosphodiesterase 5A prevents and reverses cardiac hypertrophy. Nat Med 2005; 11:214–222. 15665834 10.1038/nm1175

[bib68] Berry JM, Le V, Rotter D, et al. Reversibility of adverse, calcineurin-dependent cardiac remodeling. Circ Res 2011; 109:407–417. 21700928 10.1161/CIRCRESAHA.110.228452 PMC3164792

[bib69] Fagard RH, Celis H, Thijs L, Wouters S. Regression of left ventricular mass by antihypertensive treatment: a meta-analysis of randomized comparative studies. Hypertension 2009; 54:1084–1091. 19770405 10.1161/HYPERTENSIONAHA.109.136655

[bib70] Vega RB, Konhilas JP, Kelly DP, Leinwand LA. Molecular mechanisms underlying cardiac adaptation to exercise. Cell Metab 2017; 25:1012–1026. 28467921 10.1016/j.cmet.2017.04.025 PMC5512429

