# Supplementary material for: Cardiotrophin 1 stimulates beneficial myogenic and vascular remodeling of the heart
Source: Cell Res. 2017 Aug 8;27(10):1195–215. doi: 10.1038/cr.2017.87 (PMC5630684; doi:10.1038/cr.2017.87)
Supplement: Supplementary information, Figure S1 — hCT1 engages a restricted activation of the intrinsic caspase-mediated cell death pathway. [file cr201787x1.pdf]

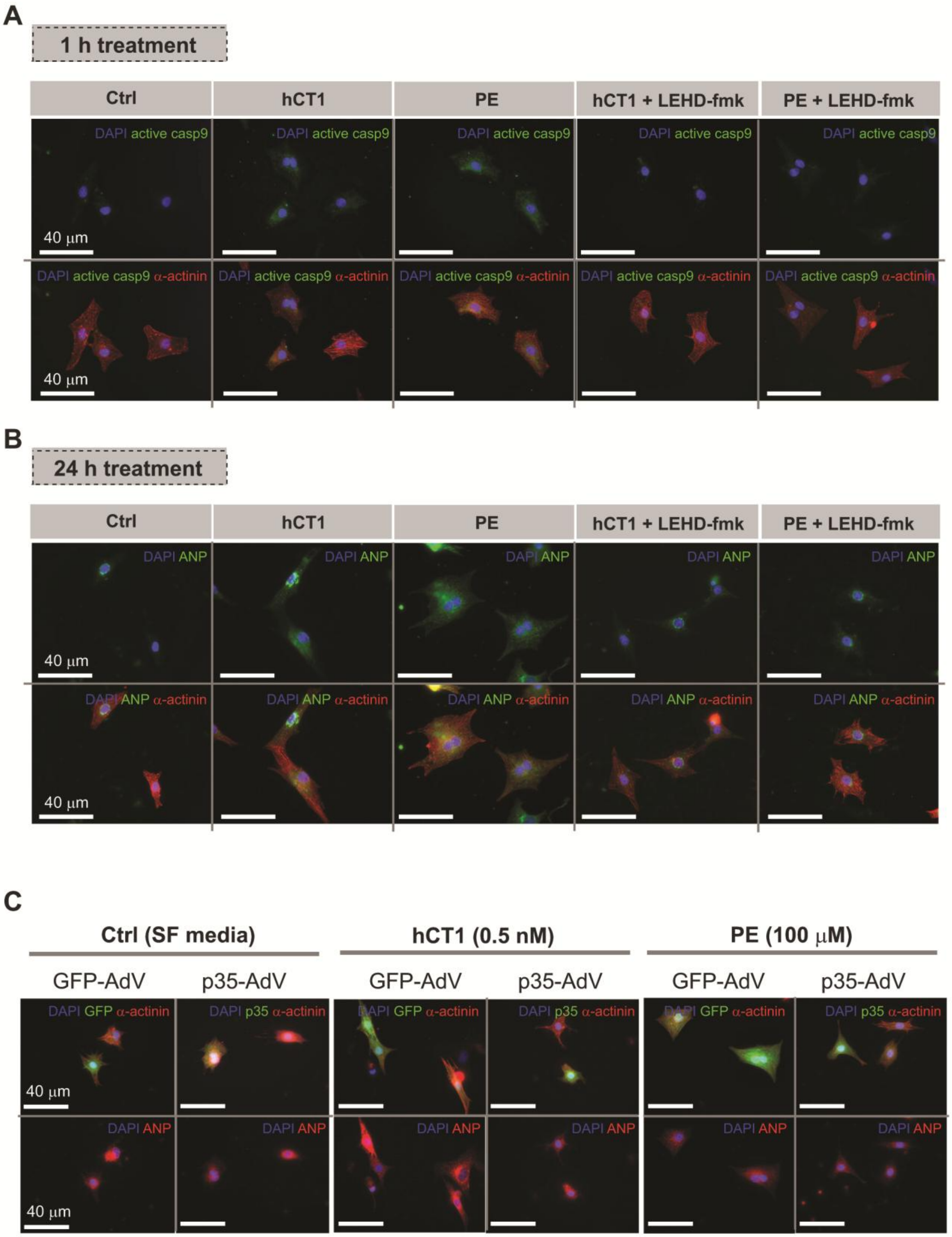
A
1 h treatment
Ctrl
hCT1
PE
hCT1 + LEHD-fmk
PE + LEHD-fmk
DAPI active casp9
DAPI active casp9 α-actinin
40 μm
B
24 h treatment
DAPI ANP
DAPI ANP α-actinin
C
Ctrl (SF media)
hCT1 (0.5 nM)
PE (100 μM)
GFP-AdV
p35-AdV
DAPI GFP α-actinin
DAPI p35 α-actinin
DAPI ANP

Supplementary information, Figure S1. hCT1 engages a restricted activation of the intrinsic caspase-mediated cell death pathway. (A-B) Immunocytochemistry images from Figure 3A-B (1 h treatment) and Figure 3E-F (24 h treatment). Primary rat cardiomyocytes were treated for 1 h (Panel A) or 24 h (Panel B) with control serum-free medium (Ctrl), hCT1 (0.5 nM), or PE (100 μM) in the presence or absence of the caspase 9 peptide inhibitor (z-LEHD-fmk, 20 μM). Immunocytochemistry was used to stain cells for: α-actinin (red), active caspase 9 (green; for 1 h treatment), the pro-hypertrophic marker atrial natriuretic peptide – ANP (green; for 24 h treatment), and nuclei were stained with DAPI (blue). Scale bar, 40 µm. (C) Immunocytochemistry images from Figure 3G-H. Cardiomyocytes were infected for 24 h with an adenovirus (AdV) encoding the biologic effector caspase inhibitor p35 upstream of a GFP reporter (p35-AdV) prior to inducing hypertrophy with hCT1 or PE for a further 24 h. A GFP only reporter (GFP-AdV) was used as a control. Immunocytochemistry was used to stain cells for: α-actinin (red), and nuclei were stained with DAPI (blue). The GFP reporter identified the positively infected cardiomyocytes (green). Scale bar, 40 µm.
