# Supplementary material for: Cardiotrophin 1 stimulates beneficial myogenic and vascular remodeling of the heart
Source: Cell Res. 2017 Aug 8;27(10):1195–215. doi: 10.1038/cr.2017.87 (PMC5630684; doi:10.1038/cr.2017.87)
Supplement: Supplementary information, Figure S2 — Pro-hypertrophic stimulation with hCT1 causes significant Stat3 activation. [file cr201787x2.pdf]

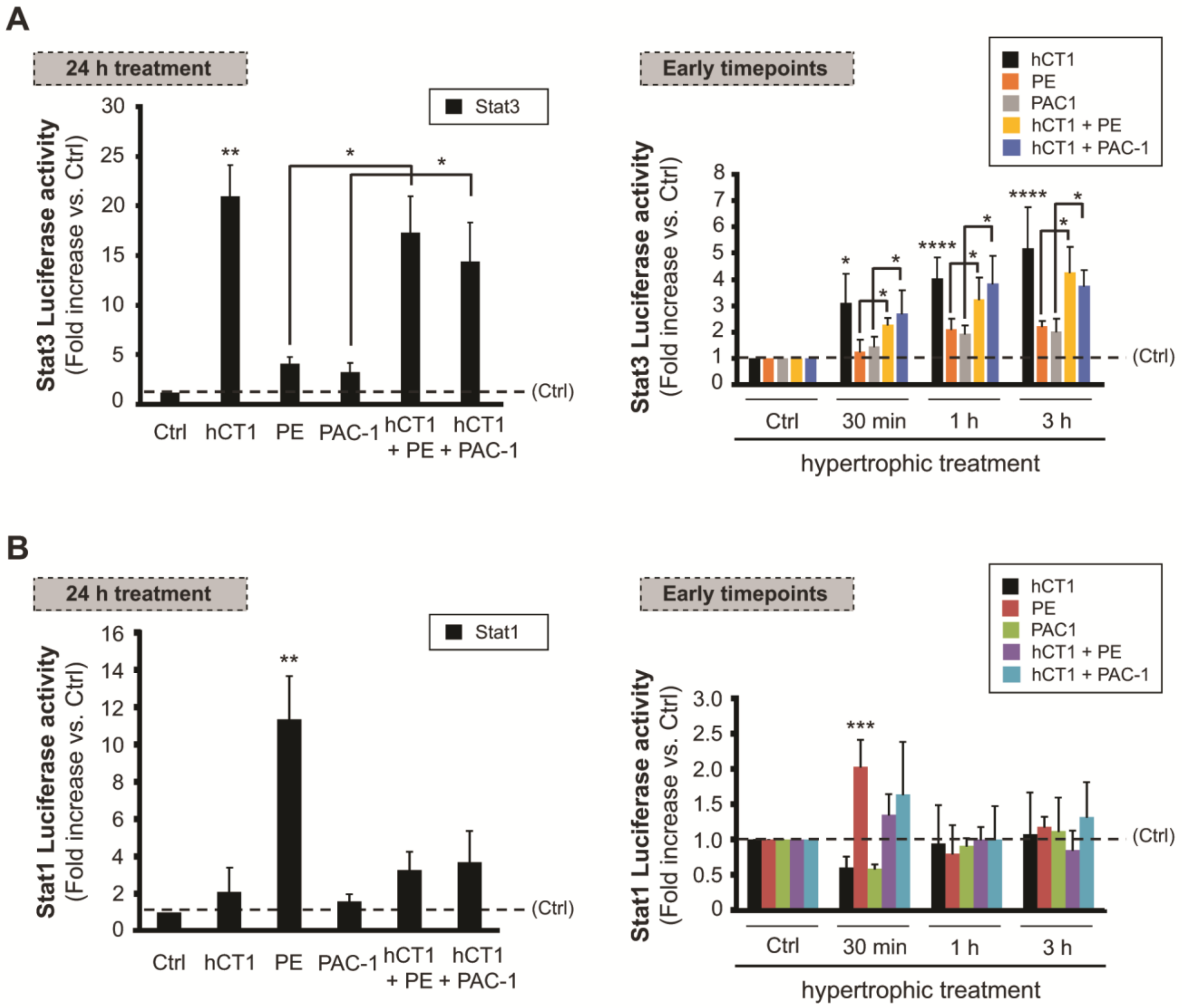
A
24 h treatment
Stat3
Stat3 Luciferase activity
(Fold increase vs. Ctrl)
Ctrl
hCT1
PE
PAC-1
hCT1 + PE
hCT1 + PAC-1
(Ctrl)
Early timepoints
hCT1
PE
PAC1
hCT1 + PE
hCT1 + PAC-1
30 min
1 h
3 h
hypertrophic treatment
B
24 h treatment
Stat1
Stat1 Luciferase activity
(Fold increase vs. Ctrl)
Early timepoints
hypertrophic treatment

Supplementary information, Figure S2. Pro-hypertrophic stimulation with hCT1 causes significant Stat3 activation. (A) Primary rat cardiomyocytes were transfected with the pro-survival Stat3 luciferase reporter plasmid and Stat3 reporter activity was measured after 24 h of treatment with: control serum-free medium (Ctrl), hCT1 (0.5 nM), PE (100 μM), or procaspase 3 activating compound 1 (PAC-1; 25 μM). Treatments were also conducted at early time points (30 min, 1 h, and 3 h) where the respective treatments for 0 min in control serum-free medium were used as control (Ctrl). hCT1 treatment significantly increased Stat3 activation at all time points when compared to control, Ctrl (n=4; *P<0.05, **P<0.01 and ****P<0.0001). Minimal Stat3 activation was observed during pathologic stimulation with PE or PAC-1. Stat3 activation was significantly increased when PE or PAC-1 were co-stimulated with hCT1 when compared to PE or PAC-1 alone, respectively. (n=4; *P<0.05). (B) Similar procedure as in (A) above, however, the pro-inflammatory Stat1 luciferase reporter plasmid was used. No significant activation of Stat1 was observed during hCT1 stimulation at any of the time points tested, however, pathologic stimulation with PE caused a significant increase in Stat1 activity at 30 min and 24 h (n=4; ***P<0.001 and **P<0.01 versus Ctrl, respectively).
