# Supplementary material for: Cardiotrophin 1 stimulates beneficial myogenic and vascular remodeling of the heart
Source: Cell Res. 2017 Aug 8;27(10):1195–215. doi: 10.1038/cr.2017.87 (PMC5630684; doi:10.1038/cr.2017.87)
Supplement: Supplementary information, Figure S3 — Casein kinase 2 (CK2) activation is required for hCT1 induced cardiomyocyte hypertrophy. [file cr201787x3.pdf]

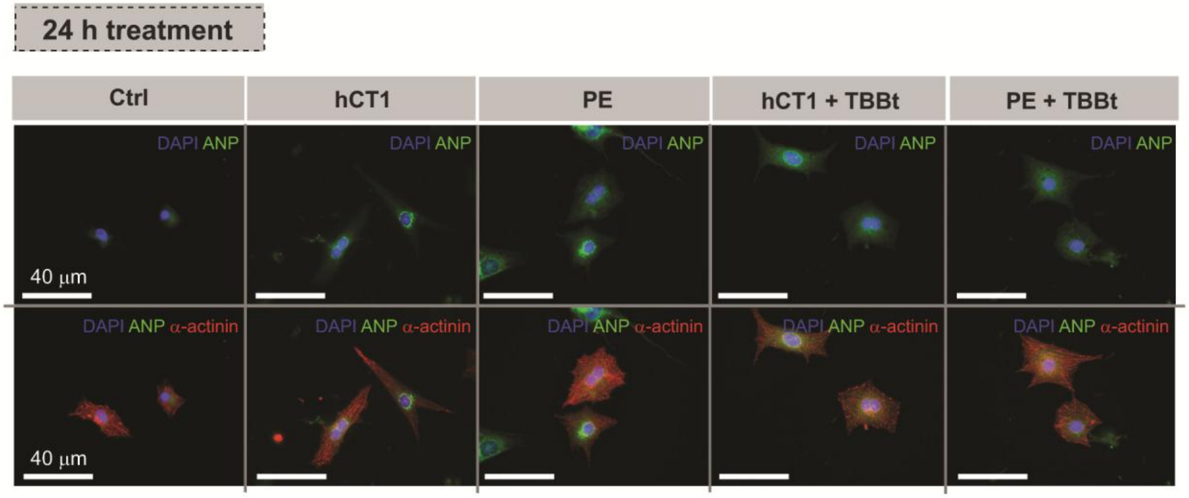
24 h treatment
Ctrl
hCT1
PE
hCT1 + TBBt
PE + TBBt
DAPI ANP
DAPI ANP α-actinin
40 μm
40 μm

Supplementary information, Figure S3. Casein kinase 2 (CK2) activation is required for hCT1 induced cardiomyocyte hypertrophy. Immunocytochemistry images from Figure 3I-K. Primary rat cardiomyocytes were treated for 24 h with control serum-free medium (Ctrl), hCT1 (0.5 nM), or PE (100 μM) in the presence or absence of the CK2 inhibitor (TBBt, 50 μM). Immunocytochemistry was used to stain cells for: α-actinin (red), the pro-hypertrophic marker atrial natriuretic peptide – ANP (green), and nuclei were stained with DAPI (blue). Scale bar, 40 μm.
