# Supplementary material for: Cardiotrophin 1 stimulates beneficial myogenic and vascular remodeling of the heart
Source: Cell Res. 2017 Aug 8;27(10):1195–215. doi: 10.1038/cr.2017.87 (PMC5630684; doi:10.1038/cr.2017.87)
Supplement: Supplementary information, Figure S4 — VEGF expression induced by hCT1 stimulation is inhibited upon blocking caspase activity. [file cr201787x4.pdf]

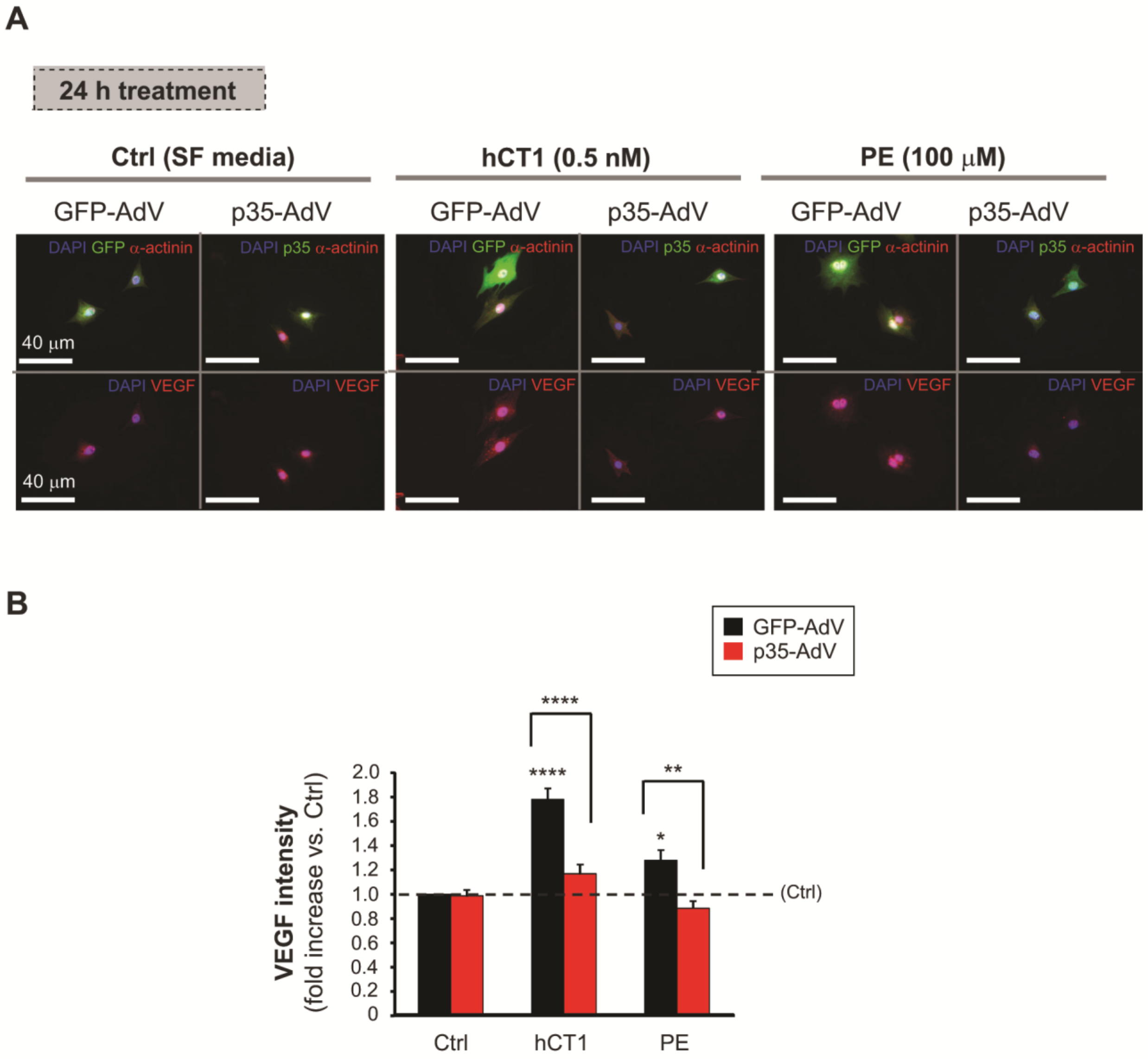
A
24 h treatment
Ctrl (SF media)
hCT1 (0.5 nM)
PE (100 μM)
GFP-AdV
p35-AdV
DAPI GFP α-actinin
DAPI p35 α-actinin
DAPI VEGF
40 μm
B
GFP-AdV
p35-AdV
VEGF intensity
(fold increase vs. Ctrl)
2.0
1.8
1.6
1.4
1.2
1.0
0.8
0.6
0.4
0.2
0
(Ctrl)
****
**
*
Ctrl
hCT1
PE

**Supplementary information, Figure S4. VEGF expression induced by hCT1 stimulation is inhibited upon blocking caspase activity.** (A) Primary rat cardiomyocytes were infected for 24 h with an adenovirus (AdV) encoding the caspase inhibitor, p35-AdV, prior to inducing hypertrophy with hCT1 or PE for 24 h. Serum-free medium was used as a control treatment (SF, Ctrl) and GFP-AdV was used as a control AdV infection. Both p35-AdV and GFP-AdV were used at a Mean of Infectivity (MOI) of 20. Immunocytochemistry was used to stain cells for: $\alpha$-actinin (far-red), VEGF (red), and nuclei were stained with DAPI (blue). The GFP reporter identified the positively infected cardiomyocytes (green). Scale bar, 40 µm. (B) Compared to control, a significant increase in VEGF expression was observed with hCT1 stimulation whereas a modest increase in VEGF was observed with PE treatment after 24 h (n=3; ****$P<0.0001$ and *$P<0.05$). In addition, p35-AdV significantly inhibited the hCT1 and PE induced increase in VEGF expression (n=3; ****$P<0.0001$ and **$P<0.01$, respectively).
