# Supplementary material for: Cardiotrophin 1 stimulates beneficial myogenic and vascular remodeling of the heart
Source: Cell Res. 2017 Aug 8;27(10):1195–215. doi: 10.1038/cr.2017.87 (PMC5630684; doi:10.1038/cr.2017.87)
Supplement: Supplementary information, Figure S5 — hCT1 stimulation increases the oxygen consumption rate of cardiomyocytes. [file cr201787x5.pdf]

Oxygen Consumption Rate

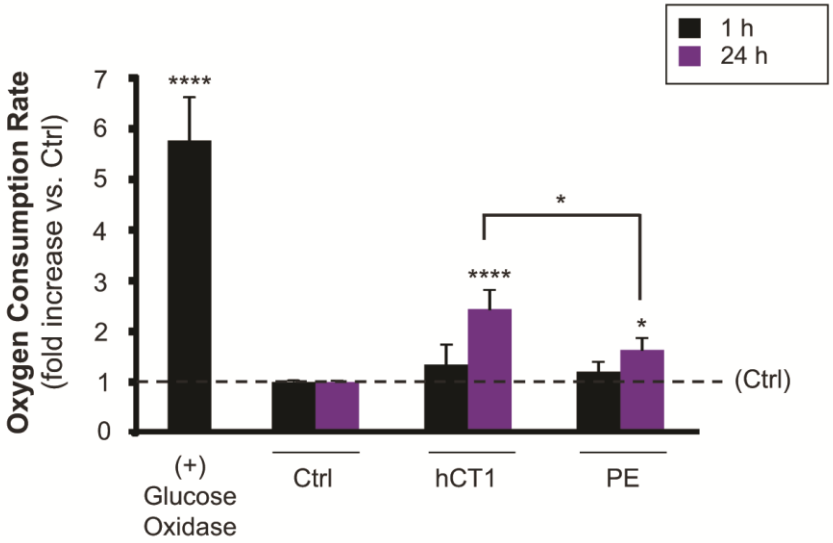

Supplementary information, Figure S5. hCT1 stimulation increases the oxygen consumption rate of cardiomyocytes. Primary rat cardiomyocytes were treated for 1 h or 24 h with hCT1 (0.5 nM) or PE (100 μM). Serum-free control medium (Ctrl) was used as a non-hypertrophic control. Glucose Oxidase enzyme treatment was used as a positive control. The phosphorescent oxygen probe (MitoXpress® - Xtra) was used to measure the rate of oxygen consumption in whole cells by standard fluorescence intensity analysis using an excitation of 380 nm and emission of 650 nm. The Glucose Oxidase positive control (+) caused a robust increase in oxygen consumption versus control at 1 h (n=3; ****P<0.0001). At 24 h treatment, hCT1 had a significant increase in oxygen consumption and PE had a modest increase versus Ctrl (n=3; ****P<0.0001 and *P<0.05, respectively). Furthermore, hCT1 had a significantly higher rate of oxygen consumption versus pathologic stimulation with PE at 24 h treatment (n=3; *P<0.05).
