# Supplementary material for: Cardiotrophin 1 stimulates beneficial myogenic and vascular remodeling of the heart
Source: Cell Res. 2017 Aug 8;27(10):1195–215. doi: 10.1038/cr.2017.87 (PMC5630684; doi:10.1038/cr.2017.87)
Supplement: Supplementary information, Figure S6 — hCT1 stimulation of cardiac fibroblasts does not significantly induce expression of the pro-fibrotic marker Galectin-3. [file cr201787x6.pdf]

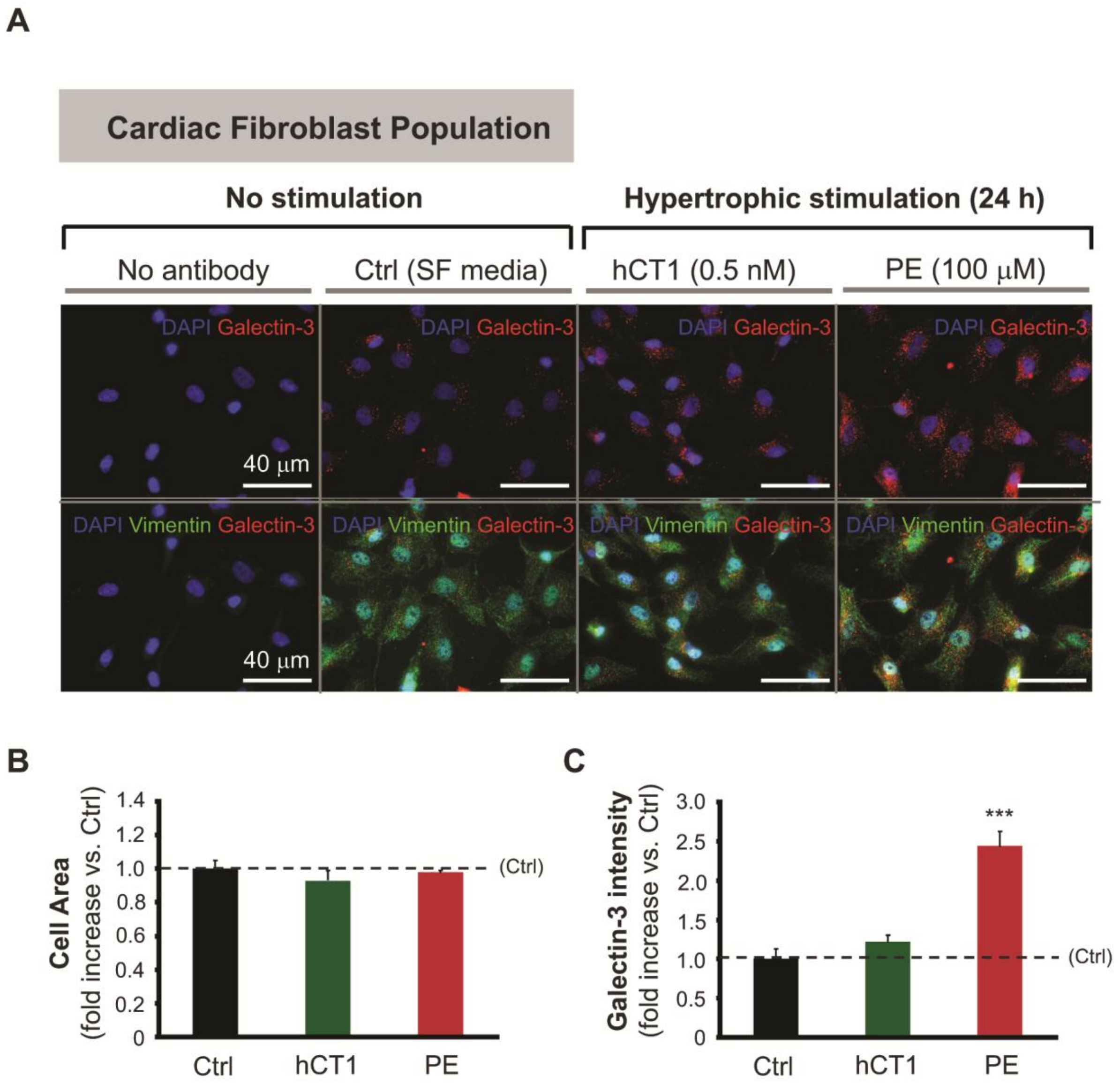

A
Cardiac Fibroblast Population
No stimulation
Hypertrophic stimulation (24 h)
No antibody
Ctrl (SF media)
hCT1 (0.5 nM)
PE (100 μM)
DAPI Galectin-3
DAPI Vimentin Galectin-3
40 μm
B
Cell Area
(fold increase vs. Ctrl)
1.4
1.2
1.0
0.8
0.6
0.4
0.2
0
(Ctrl)
Ctrl
hCT1
PE
C
Galectin-3 intensity
(fold increase vs. Ctrl)
3.0
2.5
2.0
1.5
1.0
0.5
0
***
(Ctrl)
Ctrl
hCT1
PE

Supplementary information, Figure S6. hCT1 stimulation of cardiac fibroblasts does not significantly induce expression of the pro-fibrotic marker Galectin-3. (A) The cardiac fibroblast population of cells (derived from the pre-plating step of the primary cardiomyocyte isolation procedure) were incubated with control serum-free medium (Ctrl, SF medium), hCT1 (0.5 nM), or PE (100 μM) for 24 h. Immunofluorescence was used to detect the cardiac fibroblast marker Vimentin (green), the pro-fibrotic marker Galectin-3 (red), and nuclei were stained with DAPI (blue). The control without primary antibody is devoid of non-specific staining. Scale bar, 40 μm. (B-C) Pathologic stimulation with PE resulted in a significant increase in the expression of Galectin-3 versus control (n=3; ***$P<0.001$) whereas hCT1 did not significantly enhance pro-fibrotic Galectin-3 expression versus non-stimulated control cells. Cell area of cardiac fibroblasts was not significantly changed with hCT1 or PE treatments when compared to control.
