# Supplementary material for: Cardiotrophin 1 stimulates beneficial myogenic and vascular remodeling of the heart
Source: Cell Res. 2017 Aug 8;27(10):1195–215. doi: 10.1038/cr.2017.87 (PMC5630684; doi:10.1038/cr.2017.87)
Supplement: Supplementary information, Figure S7 — hCT1 attenuates the effects observed upon inducing myocardial infarction (MI) by ligation of the Left Anterior Descending (LAD) coronary artery. [file cr201787x7.pdf]

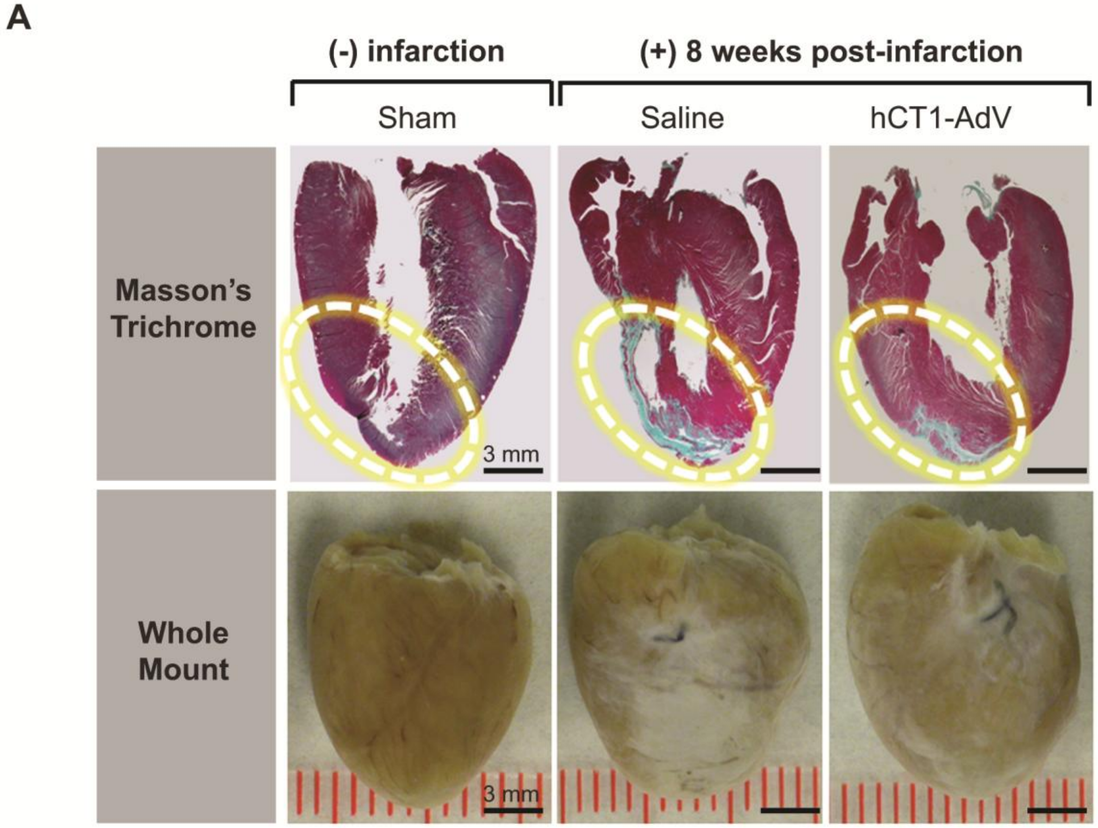
A
(-) infarction
(+) 8 weeks post-infarction
Sham
Saline
hCT1-AdV
Masson's Trichrome
Whole Mount
3 mm
3 mm

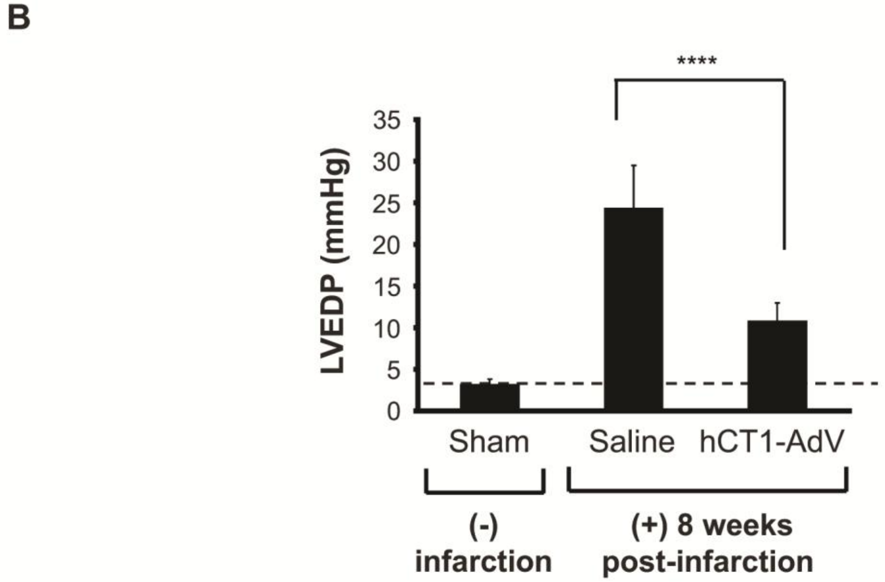
B
****
LVEDP (mmHg)
35
30
25
20
15
10
5
0
Sham
Saline
hCT1-AdV
(-) infarction
(+) 8 weeks post-infarction

Supplementary information, Figure S7. hCT1 attenuates the effects observed upon inducing myocardial infarction (MI) by ligation of the Left Anterior Descending (LAD) coronary artery. (A) Rats were subjected to LAD ligation surgery followed by intracardiac injection of hCT1 Adenovirus (hCT1-AdV at 7.5 x 10^5 infectious particles per gram body weight) or of saline. A separate group of control animals were sham operated (open cavity, no LAD). Animals were given a booster injection at 2 weeks following surgery. At 8 weeks post-MI, the left ventricle end-diastolic pressure (LVEDP) was measured using a 2F high-fidelity micro-manometer catheter (Millar Institute). Rats were then euthanized and whole hearts fixed in 10% formalin for immunohistologic analysis. Cross-sections (5-8 μm) were stained with Masson's Trichrome to visualize fibrosis in the infarct area. A significant decrease in fibrosis/collagen deposition was observed in the hCT1-treated animals versus saline as visualized by Whole mount (white-coloured scar tissue) and Masson's Trichrome (blue-coloured scar tissue). Scale bar, 3 mm. (B) A significant improvement in hemodynamic cardiac function and ventricular performance was noted as there was a significant decrease in LVEDP in the hCT1 treated animals versus saline (n=5, ****P<0.0001).
