# Supplementary material for: Cardiotrophin 1 stimulates beneficial myogenic and vascular remodeling of the heart
Source: Cell Res. 2017 Aug 8;27(10):1195–215. doi: 10.1038/cr.2017.87 (PMC5630684; doi:10.1038/cr.2017.87)
Supplement: Supplementary information, Table S1 — Microarray analysis of hCT1 stimulated primary cardiomyocytes. [file cr201787x8.pdf]

	Gene name	Gene symbol	Accession #	Fold change (vs. Ctrl) *
	**Angiogenesis & Endothelial Growth/Survival Factors**			
	Angiopoietin-like 4	Angptl4	NM_199115	2.71
	Fms-related tyrosine kinase 4	Flt4	NM_053652	2.26
	Von Willebrand factor 5A	Vwa5a	NM_198755	2.03
	Vascular Endothelial Growth Factor A	Vegfa	NM_031836	1.14
	**Antimicrobial Peptides**			
	Regenerating islet-derived 3 gamma	Reg3g	NM_173097	28.39
	Regenerating islet-derived 1 alpha	Reg1a	NM_012641	7.96
	Regenerating islet-derived 3 beta	Reg3b	NM_053289	6.14
	Hepcidin Antimicrobial Peptide	Hamp	NM_053469	2.70
	**Pro-survival Receptors**			
	Phosphoinositide-3-kinase, subunit 1, alpha	Pik3r1	NM_013005	1.56
	Phosphoinositide-3-kinase, subunit 6	Pik3r6	NM_001081444	1.49
	**Interferons**			
	Immunity-related GTPase family, M	Irgm	NM_001012007	0.78
	Interferon gamma	Ifng	NM_138880	n.s.
	Interferon regulatory factor 7	Irf7	NM_001033691	0.55
	Interferon-induced protein 44	Ifi44	NM_001107729	0.64
	Interferon-induced protein 35	Ifi35	NM_001009625	0.70
	Interferon, alpha-inducible protein 27 like 2B	Ifi27l2b	NM_206846	0.66
	Interferon beta 1	Ifnb1	NM_019127	n.s.
	Interferon stimulated exonuclease gene 20	Isg20	NM_001008510	0.76
	**Collagens**			
	Collagen, type IV, alpha 3	Col4a3	NM_001135759	0.65
	Collagen, type IV, alpha 1	Col4a1	NM_001135009	0.88
	Collagen, type IV, alpha 4	Col4a4	NM_001008332	0.74
	Collagen, type VIII, alpha 1	Col8a1	NM_001107100	0.86
	**Anti-oxidants**			
	Superoxide dismutase 2, mitochondrial	Sod2	NM_017051	1.27
	Metallothionein 2A	Mt2A	NM_001137564	1.98
	**Mitochondrial Energy Metabolism (Complexes I-V)**			
I	NADH dehydrogenase (ubiquinone) 1 alpha subcomplex, 1	Ndufa1	NM_001108813	0.75
	NADH dehydrogenase (ubiquinone) 1 alpha subcomplex, 2	Ndufa2	NM_001106153	0.74
	NADH dehydrogenase (ubiquinone) 1 alpha subcomplex 5	Ndufa5	NM_012985	0.79
	NADH dehydrogenase (ubiquinone) 1 alpha subcomplex, 8	Ndufa8	NM_001047862	0.82
	NADH dehydrogenase (ubiquinone) 1 alpha subcomplex 10	Ndufa10	NM_199495	0.77
II	Succinate dehydrogenase complex, subunit B, iron sulfur (Ip)	Sdhb	NM_001100539	0.77
	Succinate dehydrogenase complex, subunit D, integral protein	Sdhd	NM_198788	0.88
III	Cytochrome c-1	Cyc1	NM_001130491	0.89
	Ubiquinol-cytochrome c reductase core protein I	Uqcrc1	NM_001004250	0.88
	Ubiquinol-cytochrome c reductase, complex III subunit VII	Uqcrq	NM_001025134	0.81
	Ubiquinol-cytochrome c reductase, complex III subunit XI	Uqcr11	NM_001126097	0.85
IV	Cytochrome c oxidase subunit IV isoform 1	Cox4i1	NM_017202	0.82
	Cytochrome c oxidase subunit VIIb	Cox7b	NM_182819	0.79
	Cytochrome c oxidase, subunit VIIc	Cox7c	NM_001134705	0.86
	Cytochrome c oxidase, subunit VIIIb	Cox8b	NM_012786	0.56
V	ATP synthase, mitochondrial F1 complex, alpha 1, cardiac	Atp5a1	NM_023093	0.85
	ATP synthase, mitochondrial F1 complex, epsilon	Atp5e	NM_139099	0.87
	ATP synthase, mitochondrial Fo complex, subunit E	Atp5i	NM_080481	0.73
	ATP synthase, mitochondrial Fo complex, subunit F6	Atp5j	NM_053602	0.82

Supplementary information, Table S1. Microarray analysis of hCT1 stimulated primary cardiomyocytes. Primary cardiomyocytes were stimulated with hCT1 (0.5 nM) for 24 h followed by isolation of total RNA which was then subjected to expression profiling analysis by Microarray (Affymetrix Rat GeneChip 1.0ST). Serum-free medium was used as a control (Ctrl). Significant differences in gene expression were observed when compared to control (n=3; *P<0.05) while some genes had no significant difference (n.s.) in gene expression. Some of the protein family groups of target genes activated were: angiogenesis and endothelial growth/survival factors, antimicrobial peptides, pro-survival receptors, and anti-oxidants. Some of the target genes families that decreased were: collagens, interferons, and mitochondrial energy metabolism.
